# Phytohormonal and Transcriptomic Mechanisms of Multigenerational Stress Memory in Wheat Under Weed Competition

**DOI:** 10.1111/pce.70475

**Published:** 2026-03-11

**Authors:** Albert O. Kwarteng, Joseph C. Kuhl, Brenda M. Murdoch, Fangming Xiao, Albert T. Adjesiwor

**Affiliations:** ^1^ Department of Plant Sciences, Kimberly Research and Extension Center University of Idaho Kimberly Idaho USA; ^2^ Present address: College of Agriculture, Environmental, and Human Sciences Lincoln University of Missouri Jefferson City Missouri USA; ^3^ Department of Plant Sciences University of Idaho Moscow Idaho USA; ^4^ Department of Animal, Veterinary & Food Sciences University of Idaho Moscow Idaho USA

**Keywords:** adaptive and maladaptive responses, biotic stress, crop‐weed competition, differential gene expression, hormones, phytohormones, stress memory response, transcriptome, weed competition

## Abstract

Multigenerational stress exposure induces stress memory in plants, influencing resource allocation, defence mechanisms, and productivity. Weed competition imposes both resource‐based (abiotic) and allelopathic (biotic) stress, engaging overlapping hormonal pathways. This study examined the hormonal and transcriptomic mechanisms underlying multigenerational stress memory in wheat subjected to inter‐specific competition with kochia and Italian ryegrass and intra‐specific competition with other wheat plants. Phytohormone analysis revealed increased salicylic acid levels, promoting systemic acquired resistance, whereas jasmonic acid levels declined, indicating suppressed jasmonate‐mediated defence. Abscisic acid responses varied, reflecting shifts in water‐use efficiency. Cytokinins and auxins exhibited generation‐ and treatment‐specific trends, suggesting adaptive resource acquisition but potential hormonal imbalances. These hormonal shifts corresponded with phenotypic responses, where adaptive benefits peaked at Generation 3 before transitioning to maladaptive responses in later generations. Transcriptomic analysis identified dynamic changes in differentially expressed genes (DEGs) and key pathways. Wheat‐only competition peaked in stress‐responsive DEGs in Generation 3, while wheat‐kochia and wheat‐ryegrass exhibited early generation transcriptional reprogramming and long‐term adaptations. Intra‐specific wheat competition showed early generation transcriptomic surges but persistent growth repression in the current study. These findings provide mechanistic insights into multigenerational stress memory mechanisms and reveal how phytohormonal crosstalk and transcriptional reprogramming shape wheat responses to competition stress across generations.

## Summary statement

Multigenerational competition in *Triticum aestivum* triggered treatment‐specific shifts in hormone signalling and gene expression, revealing both adaptive and maladaptive stress‐memory responses that emerged at different generations under prolonged biotic stress.

## 1 Introduction

Plants face numerous biotic and abiotic stressors that require sophisticated adaptive responses for survival and productivity. Weed competition is a major biotic stress that reduces resource availability and causes significant yield losses (Oerke 2006; Chauhan 2020). Weed competition is a significant biotic stress on crops because weeds share a similar trophic level and can become highly competitive, aggressive, and troublesome (Smith and Burns 2022). For instance, weeds such as kochia (*Bassia scoparia*) and Italian ryegrass (*Lolium multiflorum*) are particularly aggressive competitors of wheat (*Triticum aestivum*), with yield reductions reaching 73% in spring wheat and 92% in winter wheat (Nandula 2014; Nair et al. 2021). Weed competition differs from classical biotic stresses in several key ecological and physiological aspects. While classical biotic stresses typically include pests and pathogens that cause direct damage or disease symptoms, weed competition operates through different mechanisms. Weed competition imposes stress primarily through resource competition for light, water, nutrients, and space, processes mechanistically analogous to abiotic stresses, and secondarily through allelopathic interference via secondary metabolite release (Savić et al. 2025), mechanisms more analogous to abiotic stress. This dual nature engages overlapping stress response pathways, where resource limitation activates abiotic stress hormones, while allelopathy and competitive signalling may trigger biotic defence responses (Verma et al. 2016; Shan et al. 2023). Understanding how multigenerational exposure integrates these responses is critical for developing competitive crop varieties.

Recent research suggests that multigenerational stress priming, in which plants exposed to stress in one generation demonstrate increased resilience in subsequent generations, may offer a promising avenue for minimising chemical inputs in agriculture (Conrath et al. 2015; Prashant Singh and Roberts 2015). By activating intrinsic defence mechanisms, stress priming induces stress memory, enabling plants to “remember” prior stress events and adjust their responses to future challenges (Fleta‐Soriano and Munné‐Bosch 2016; Kambona et al. 2023). This form of plant protection, which involves activating intrinsic defence mechanisms across generations (Ramírez‐Carrasco et al. 2017), could contribute to more sustainable agricultural practices by minimising herbicide use. Stress memory is mediated by transcriptional reprogramming, epigenetic changes, and hormonal signalling, enabling plants to enhance their defence mechanisms or conserve resources (Hilker et al. 2016). However, the distinction between adaptive stress memory (enhanced performance upon re‐exposure) and maladaptive responses (fitness costs from prolonged or mismatched stress) remains poorly understood, particularly across extended generational timescales. Although adaptive memory may enhance stress tolerance and resource acquisition prolonged stress exposure may also lead to maladaptive responses, such as reduced photosynthetic efficiency, delayed recovery, and diminished growth, which compromise long‐term productivity (Walter et al. 2013; Crisp et al. 2016; Hilker et al. 2016).

Despite the growing body of literature on multigenerational stress priming, there remains a notable gap in research on its effects on crop‐weed interactions (Kwarteng et al. 2025). This is concerning, considering that weeds cause substantial crop yield losses, ranging from 10% to 98% depending on the crop and region (Oerke 2006; El‐Dabaa et al. 2022). Exploring the role of stress priming in mitigating these losses could provide insights into the development of sustainable agricultural practices. Moreover, different weed species impose distinct competitive pressures through varied resource‐use strategies and/or allelopathic capacities; however, how crops integrate and ‘remember’ these multifaceted stresses across generations remains unexplored.

Phytohormones, such as abscisic acid (ABA), jasmonic acid (JA), and salicylic acid (SA), serve as key integrators of stress responses, with distinct hormones mediating different aspects of competitive stress. ABA regulates stomatal closure and root growth under water and nutrient limitations (Verma et al. 2016), responses directly relevant to resource competition with weeds. Auxins and cytokinins govern root architecture and nutrient acquisition, enabling plants to forage more effectively in competitive environments (Jan et al. 2024). Meanwhile, JA and SA mediate defence responses to biotic challenges, including neighbour detection and allelopathic stress (Shan et al. 2023). The crosstalk between these pathways reflects the integrated nature of weed competition, where plants must simultaneously manage resource scarcity (abiotic‐like) and chemical interference (biotic‐like). Research has shown that these phytohormones also contribute to multigenerational stress priming, shaping both immediate and inherited responses (Ding et al. 2012; Hilker et al. 2016; Kambona et al. 2023). For example, JA signalling pathways regulate resource allocation under competitive stress, whereas SA enhances systemic acquired resistance (SAR) (Li et al. 2022).

Advances in transcriptomics have provided new opportunities to investigate the molecular mechanisms underlying stress memory. Differential gene expression studies have revealed key pathways involved in stress responses, including hormonal signalling, structural reinforcement, and metabolic adaptation (Chaffai et al. 2024). Transcription factors such as Ethylene Response Factor (ERF), Nam‐Ataf‐Cuc (NAC), WRKY, and Myeloblastosis (MYB)‐related proteins regulate these pathways, mediating the trade‐offs between growth and defence (Rai et al. 2024). For instance, whereas NAC transcription factors play a crucial role in plant immunity by regulating defence genes and strengthening cell walls, WRKY transcription factors are pivotal in mediating plant defence responses by activating stress‐related genes and modulating hormonal signalling pathways, particularly JA and SA responses (Dong et al. 2024). Despite these insights, the interplay between transcriptomic changes and phytohormonal dynamics in multigenerational stress memory remains poorly understood, particularly in wheat.

Wheat is one of the world's most important staple crops, serving as a significant staple food for over two billion people (Singh et al. 2020; Ramzan et al. 2023). Its adaptability and economic importance make it an ideal “model” crop for studying multigenerational crop‐weed interactions. In the United States, wheat ranks third in terms of production value among field crops, behind corn and soybeans (Igrejas and Branlard 2020), making it a key component of sustainable agricultural systems. Studies have shown that rotating wheat with other crops can reduce weed density and maintain species diversity, preventing the domination of problematic weeds and enhancing soil health (Kanatas 2020). However, the potential of multigenerational stress priming to enhance wheat resilience to weed competition and other biotic stressors remains largely unexplored. Addressing this gap could contribute to the development of more resilient wheat cultivars.

In a previous study, wheat exposed to five generations of weed or wheat competition exhibited both potentially maladaptive and beneficial effects on wheat yield and biomass traits (Kwarteng et al. 2025). To elucidate the mechanisms underlying these phenotypic responses, we investigated hormonal and transcriptomic changes across three biologically distinct competition scenarios. Kochia is an aggressive C4 weed with deep taproots, high drought tolerance, and documented allelopathic capacity (Nair et al. 2021), whereas Italian ryegrass is a fast‐growing C3 competitor that rapidly depletes soil nitrogen and shades wheat canopies (Nandula 2014). These interspecific competitors represent distinct resource use strategies and chemical interference mechanisms. In contrast, intra‐specific competition among wheat provides a comparison for neighbour presence and density stress in the absence of weed‐specific traits such as allelopathy or divergent resource acquisition strategies, allowing us to distinguish weed‐specific stress memory from general crowding responses. By integrating phytohormonal quantification and transcriptomic profiling, we aimed to elucidate how wheat adapts to multigenerational competition stress. Specifically, this study addressed the following objectives:

1. Characterise changes in key phytohormones such as ABA, JA, SA, the auxin, Indole‐3‐Acetic Acid (IAA), cytokinins, cis‐ and trans‐Zeatin (cZ and tZ), cis‐ and trans‐Zeatin Riboside (cZR and tZR), and JA‐related compounds, Jasmonoyl‐Isoleucine (JA‐Ile), 12‐Oxo‐Phytodienoic Acid (OPDA) in multigenerational crop‐weed interactions.
2. Assess differentially expressed genes (DEGs) and key functional pathways associated with multigenerational crop‐weed interactions.
3. Explore the interplay between hormonal regulation and transcriptional reprogramming in multigenerational crop‐weed interactions.

## 2 Results

### 2.1 Phytohormonal Dynamics Across Treatments and Generations

In a previous study, we reported potential maladaptive effects of multigenerational stress memory on wheat yield and biomass after five generations of weed or wheat competition (Kwarteng et al. 2025). Specifically, wheat‐only (WO) and wheat‐kochia (WK) treatments exhibited peak biomass and yield at Generation 3, followed by a decline in subsequent generations, whereas wheat‐ryegrass (WR) and wheat–wheat (WW) treatments showed variable performance across generations. The current study revealed significant phytohormone changes across treatments and generations compared to Generation 0 (parent seed). The tested phytohormones included auxins (IAA), cytokinins (cZ, cZR, and tZ), jasmonates (JA, JA‐Ile, and OPDA), SA, and ABA (Table 1). SA levels increased sharply, with a 151% increase in WO Generation 2 and a 101% increase in WW Generation 1. WK and WR showed increases of 74% and 72% in Generations 2 and 3, respectively. JA and JA‐Ile consistently declined across treatments, with JA decreasing by 80% in WO Generation 2 and JA‐Ile decreasing by 85% in Generation 5. WK and WR showed similar reductions, with declines of up to 81% and 89%, respectively. In contrast, OPDA increased, with an eightfold rise in WO Generation 3 and similar trends in WK and WW, particularly in Generations 3 and 4. ABA and cytokinin trends varied. In WO, ABA peaked at 34% in Generation 5, whereas WK and WR saw declines of up to 31% and 43%, respectively, suggesting suppressed ABA signalling under weed competition. Cytokinin levels in WO showed a 171% tZ increase in generation 1% and 77% in Generation 3, followed by decline. WK saw increases in all cytokinins in Generation 3, while WW had a 345% tZ rise in Generation 5. These results highlight substantial variation in phytohormonal regulation under multigenerational competition stress. SA and OPDA increased in specific treatments and generations, while JA and JA‐Ile consistently declined.

**Table 1:** Percentage change in phytohormone levels in wheat (Generations 1–5) compared to Generation 0 across different competition treatments.

Treatment	Generation	ABA	SA	JA	JA‐ILE	OPDA	IAA	cZ	cZR	tZ
		(%)	(%)	(%)	(%)	(%)	(%)	(%)	(%)	(%)
Wheat‐only	1	−6	18	−77	−80	473	20	27	112	171
	2	−21	151**	−80	−64	775	16	−46*	4	−3
	3	29	29	−51	−64	814	29	−43*	−62	77
	4	−27	17	−65	−53*	−29	28	−26	−52	−34
	5	−34	26	−71	−85	161	36	23	−71	−30
Wheat‐kochia	1	−28	−20	−73	−77	−45	61	−61*	−81	−11
	2	−31	74*	−36	−69	−36	36	−67**	−81	−19
	3	−30	9	−79	−80	216	−2	76	245	63*
	4	−2	34	−47	−36	11	48	−49*	−56	3
	5	−21	−1	−71	−92	250	53	−49*	−44	59*
Wheat‐ryegrass	1	−21	26*	−71	−89	−27	40	−40*	−53	129
	2	−36	21	−66	−27	−51	23	−44*	−54	−29
	3	−8	72*	−81	−86	36	25	−33	−70	211
	4	−43	34	−53	−38	63	21	−62**	−89	163
	5	−41	50*	−37	−56	−74	59	−48*	−77	−38
Wheat–wheat	1	−47	101**	−73	−77	−71	57	−52*	−67	−20
	2	−3	41*	−23	−1	153	31	−37	−44	204
	3	−1	−11	−17	−46	88	17	−46*	−56	421
	4	−50	10	−79	−51	67	72	−69**	−94	−16
	5	−36	56*	−79	−85	−15	46	−44*	−53	345

*Note:* Significance codes: ***p ≤ 0.001; **p ≤ 0.01; *p ≤ 0.05; no significance star *p* ≥ 0.05.

### 2.2 Transcriptome Sequencing and Assembling

Sample information for the sequencing experiment, detailing sample identification, treatment name, and corresponding treatment and generation codes, are presented in Supporting Information S1: Table S1. Raw image data from high‐throughput Illumina NovaSeq. 6000 platforms were processed using CASAVA base calling software, converting them into sequenced reads stored in FASTQ files, which included both the read sequences and quality scores. A total of 168 cDNA libraries were constructed, resulting in 8 556 666 686 raw reads. Following data filtering, 813 580 414 clean reads and 1220.35 Gb of clean bases were obtained, with each library yielding between 5.29 and 18.62 Gb of clean bases, as detailed in Supporting Information S1: Table S2. More than 90% of the clean reads from all samples were aligned to the reference genome (ensembl_56_triticum_aestivum_iwgsc_toplevel), as shown in Supporting Information S1: Table S3. Read distribution analysis indicated that the majority (> 80%) of aligned reads mapped to exonic regions across all samples, whereas intergenic regions accounted for 12%–16% of the reads, and introns accounted for 2%–4%, as shown in Supporting Information S1: Figure S1.

### 2.3 Gene Function Classification and Enrichment Analysis of DEGs

**Figure 1:**
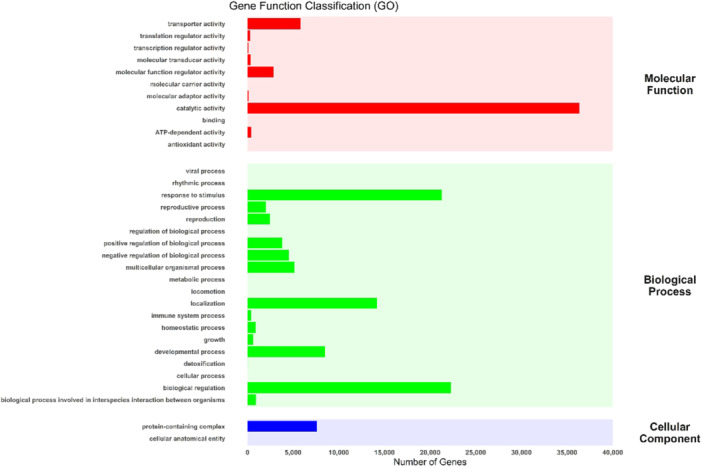
Gene function classification analysis showing the distribution of DEGs across the three Gene Ontology (GO) categories. Molecular function, biological process, and cellular component. Bars represent the total number of genes associated with each GO term, with the x‐axis denoting the number of genes. Background shading differentiates the GO categories, with molecular function (red), biological process (green), and cellular component (blue).

**Figure 2:**
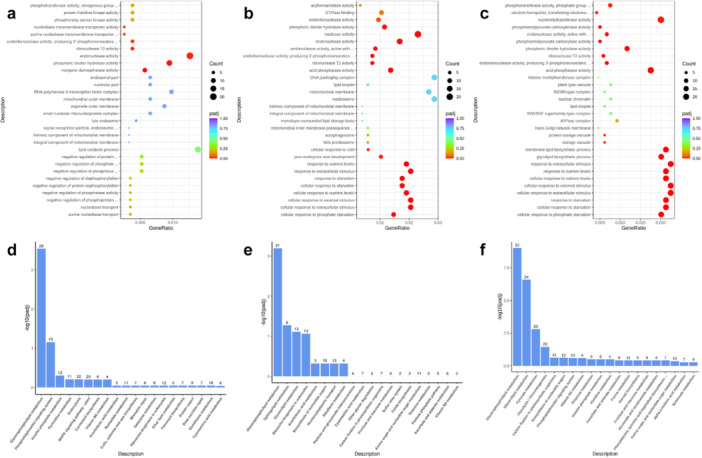
Gene Ontology (GO) enrichment analysis of wheat‐kochia, wheat‐ryegrass, and wheat–wheat treatments compared to wheat‐only (a–c) and significantly enriched pathways identified through KEGG and Reactome analyses, ranked by ‐log10 (*p*‐value) (d–f). Dot size represents the number of genes associated with each GO term, while the colour gradient indicates the significance of the adjusted *p*‐value. (a, d) wheat‐kochia, (b, e) wheat‐ryegrass, (c, f) wheat–wheat. [Color figure can be viewed at wileyonlinelibrary.com]

Transcriptome profiling of wheat leaf samples under multigenerational competition, compared to WO samples, revealed significant gene distribution across the three major GO categories: molecular function, biological process, and cellular component (Figure 1). Enrichment analysis of DEGs identified key biological processes and pathways activated in response to competition, as summarised in Figure 1. GO classifications showed shared enrichment patterns, with significant representation of stress response, photosynthesis, and metabolic regulation. Notably, oxidative stress, kinase activity, and signal transduction processes occurred in WK, WR, and WW treatments, indicating common stress adaptation mechanisms. Pathway analysis using KEGG and Reactome databases identified several enriched pathways across treatments (Figure 2). Among the shared pathways, glutathione metabolism, photosynthesis, and hormone signal transduction were highly enriched, representing core stress adaptation mechanisms. Ranked bar charts (Figure 2c–f) highlight the most significant pathways, with glycerophospholipid metabolism showing the strongest enrichment across all treatments. However, distinct patterns emerged: WK interactions showed enrichment in secondary metabolite biosynthesis, WR emphasised hormonal regulation and stress signalling, and WW treatments had pronounced enrichment in energy metabolism pathways.

### 2.4 Differential Expression of Genes

**Figure 3:**
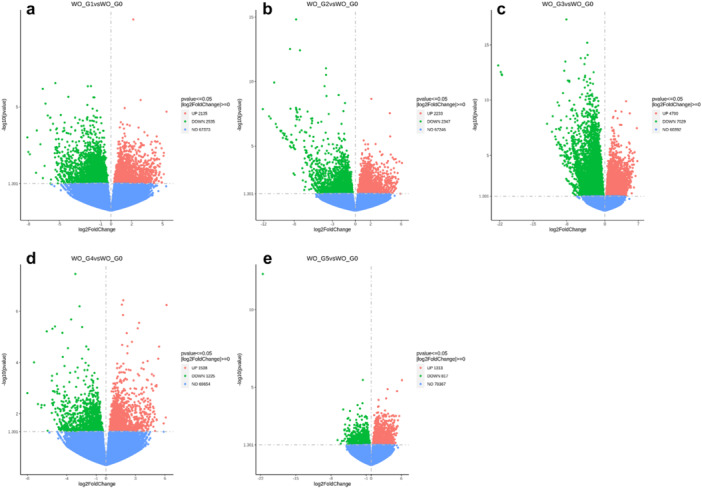
Differential expression analysis of wheat‐only treatments compared to Generation 0 wheat‐only. Volcano plots illustrate upregulated (red) and downregulated (green) genes (*p* ≤ 0.05, |log2FoldChange| ≥ 1) relative to wheat‐only control conditions. (a) Generation 1, (b) Generation 2, (c) Generation 3, (d) Generation 4, and (e) Generation 5. Non‐significant genes are shown in blue. The number of upregulated, downregulated, and non‐significant genes is indicated in the plot legends. [Color figure can be viewed at wileyonlinelibrary.com]

The number of DEGs (upregulated and downregulated) for each treatment comparison was determined using thresholds of adjusted *p*‐value (*p*adj) ≤ 0.05 and absolute log_2_ (fold change) ≥ 1 (twofold change) to identify biologically meaningful changes while controlling for false discovery rate (FDR) and visualised using volcano plots (Figure 3; Supporting Information S1: Figure S2–S4). DESeq2 analysis identified significant treatment‐ and generation‐specific variations in gene expression. In the WO treatment, the number of DEGs steadily increased from Generations 1–3, peaking at 11 729 DEGs, including 7029 upregulated and 4700 downregulated genes, indicating a strong transcriptomic response. This peak contrasts with the lower DEG counts in later generations, such as 2763 in Generation 4 and 2130 in Generation 5, reflecting a marked decline in transcriptomic activity over time (Figure 3, Supporting Information S1: Table S4). These trends suggest an early surge in differential expression, followed by possible adaptation or stabilisation in the WO treatment. Substantial transcriptomic changes also occurred in the WK treatment compared to Generation 0, particularly in later generations. While Generation 1 exhibited only 1485 DEGs, the number increased to 4555 in Generation 2 and peaked at 7045 in Generation 5, driven by 3281 upregulated and 3764 downregulated genes (Supporting Information S1: Figure S2 and Table S4). This increase suggests a heightened adaptive response to prolonged competition with Kochia.

The WR treatment showed a moderate but consistent transcriptomic response relative to Generation 0 (Supporting Information S1: Figure S3 and Table S4). Generation 1 had 2113 DEGs, nearly balanced between 1149 upregulated and 964 downregulated genes. The number increased to 3775 in Generation 3, primarily due to increased downregulation (2309 genes). In Generation 5, the DEG count declined to 2566, suggesting reduced transcriptomic activity, possibly due to long‐term adaptation to competition with ryegrass. The WW treatment exhibited distinct and variable transcriptomic responses across generations compared to Generation 0 (Supporting Information S1: Figure S4 and Table S4). Generation 1 had 9849 DEGs, with 3564 upregulated and 6285 downregulated genes. This strong response diminished in Generations 2 (2860 DEGs) and 3 (4050 DEGs) before rising again in Generation 4 (7143 DEGs). In Generation 5, the number of DEGs decreased to 1962, indicating a tapering of transcriptomic responses.

### 2.5 GO Term Enrichment Analysis of DEGs

#### 2.5.1 Wheat‐Only

**Figure 4:**
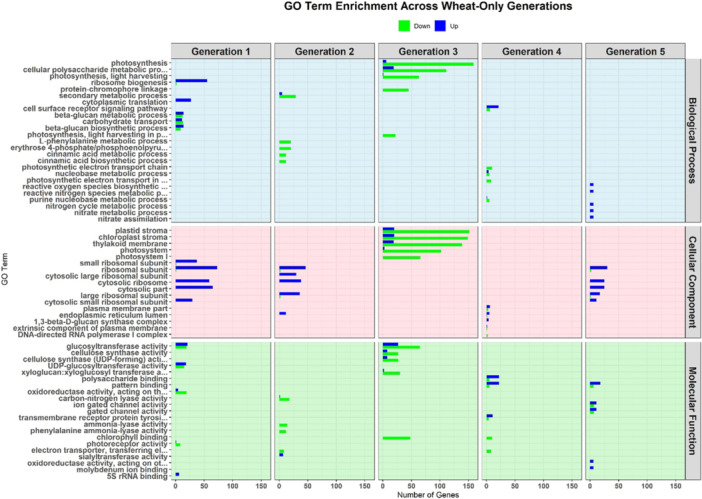
GO term enrichment analysis of differentially expressed genes for the wheat‐only treatment. Panels show changes in the Top 10 GO terms related to biological processes (light blue), cellular components (pink), and molecular functions (light green) for each generation. Upregulated genes are represented in blue, while downregulated genes are shown in green. [Color figure can be viewed at wileyonlinelibrary.com]

GO term enrichment analysis of DEGs across successive WO generations revealed shifts in key biological processes, cellular components and molecular functions. In Generation 1, ribosome biogenesis and cytoplasmic translation were upregulated, indicating enhanced protein synthesis, whereas photosynthesis and carbohydrate transport were downregulated, suggesting altered resource allocation (Figure 4). In Generation 2, L‐phenylalanine and cinnamic acid biosynthetic processes were downregulated, indicating reduced precursor production. Ribosomal subunit genes remained upregulated, supporting protein synthesis, whereas photosynthesis components remained suppressed. Generation 3 showed further downregulation of photosynthesis and cellular polysaccharide metabolism. Reductions in chloroplast stroma and thylakoid membrane components aligned with diminished photosynthetic capacity. Downregulation of glucosyltransferase and cellulose synthase genes suggested decreased cell wall remodelling. In Generation 4, the upregulation of beta‐glucan metabolic and polysaccharide binding processes indicated shifts in structural integrity and stress signalling. By Generation 5, nitrate assimilation and reactive oxygen species (ROS) biosynthesis were upregulated, reflecting increased nitrogen metabolism and stress responses. Elevated ribosomal components and pattern binding activity suggested continued support for protein production and signalling regulation.

#### 2.5.2 Wheat‐Kochia

**Figure 5:**
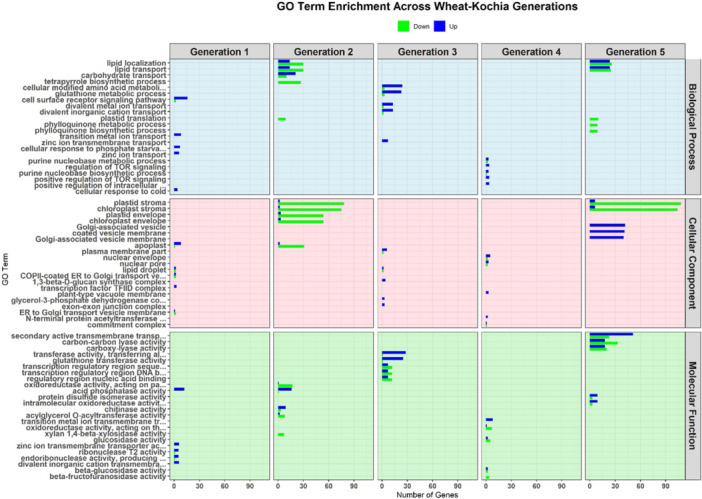
GO term enrichment analysis of differentially expressed genes for the wheat‐kochia treatment. Panels show changes in the Top 10 GO terms related to biological processes (light blue), cellular components (pink), and molecular functions (light green) for each generation. Upregulated genes are represented in blue, while downregulated genes are shown in green. [Color figure can be viewed at wileyonlinelibrary.com]

GO term enrichment analysis across WK generations revealed shifts in signalling, metabolism, and structural processes. In Generation 1, cell surface receptor signalling and metal ion transport were upregulated, indicating enhanced external signalling, along with increased acid phosphatase activity (Figure 5). In Generation 2, lipid transport, tetrapyrrole biosynthesis, and plastid stroma components were downregulated, reflecting a decline in chlorophyll‐related functions. Regulation of oxidoreductase activity showed mixed transcriptional changes, with both up‐ and downregulation. Generation 3 exhibited upregulation of glutathione metabolism and transferase activities, indicating a focus on detoxification and stress response. Meanwhile, cellular polysaccharide metabolism and photosynthesis components were downregulated. In Generation 4, TOR signalling and other intracellular regulatory processes were downregulated, suggesting reduced metabolic signalling efficiency, while polysaccharide binding remained unchanged. By Generation 5, Golgi‐associated vesicle membrane components and secondary active transmembrane transport were strongly upregulated, supporting protein transport under stress. Meanwhile, lipid and zinc ion transport were downregulated, indicating reduced lipid metabolism and micronutrient regulation (Figure 5).

#### 2.5.3 Wheat‐Ryegrass

Across WR generations, adaptive shifts progressed from early lipid and phenylpropanoid processes to enhanced photosynthetic efficiency and nutrient transport (Supporting Information S1: Figure S5). In Generation 1, ion transport and cellular responses to starvation were strongly upregulated, alongside increased acid phosphatase and endonuclease activity, indicating cellular reinforcement. Transcriptional regulation was supported by upregulated DNA‐directed RNA polymerase II and nuclear RNA polymerase components. Generation 2 showed upregulation of cell surface receptor signalling and oxylipin biosynthesis, with increased activity in the endoplasmic reticulum lumen and polysaccharide binding. Phenylpropanoid metabolism was downregulated, suggesting a shift away from secondary metabolism. In Generation 3, cell wall macromolecule metabolism was downregulated, whereas pattern binding and glutathione transferase activity were upregulated, indicating improved carbohydrate recognition and detoxification. Generation 4 exhibited increased receptor signalling and polysaccharide binding, reflecting improved environmental sensing. Lipid transport was largely downregulated, indicating stress‐driven reorganisation of cellular functions. By Generation 5, potassium and zinc ion transport were upregulated, emphasising nutrient uptake. Meanwhile, downregulation of l‐phenylalanine metabolism and carboxy‐lyase activity signalled continued metabolic reallocation to support stress tolerance (Supporting Information S1: Figure S5).

#### 2.5.4 Wheat–Wheat

The GO term enrichment analysis for WW generations highlights key shifts in metabolic and structural processes (Supplementary Figure 6). In Generation 1, cell wall and polysaccharide metabolic processes, hemicellulose metabolism, and photosynthesis were downregulated, indicating reduced structural investment and energy production. Cellular components, including photosystems I and II, were also suppressed. Generation 2 emphasised protein synthesis and stress adaptation, with upregulation of translational elongation and cellular response to starvation. Ribosomal activity was supported by transcriptional regulation associated with RNA polymerase complexes. Generation 3 showed upregulated carbohydrate transport and secondary active transmembrane transport, reflecting enhanced nutrient uptake and cellular processing. No significant structural reallocation was observed. In Generation 4, photosynthesis and phenylpropanoid metabolism were downregulated, while polysaccharide binding and pattern binding activities increased, suggesting improved sensing and cell wall interactions. Structural components like thylakoid membranes and photosystems were also suppressed. By Generation 5, key metabolic pathways remained stable with no significant upregulation observed, while transcriptional activity in chlorophyll‐binding processes remained downregulated (Supporting Information S1: Figure S6).

### 2.6 Hierarchical Clustering Heatmaps of Gene Expression

**Figure 6:**
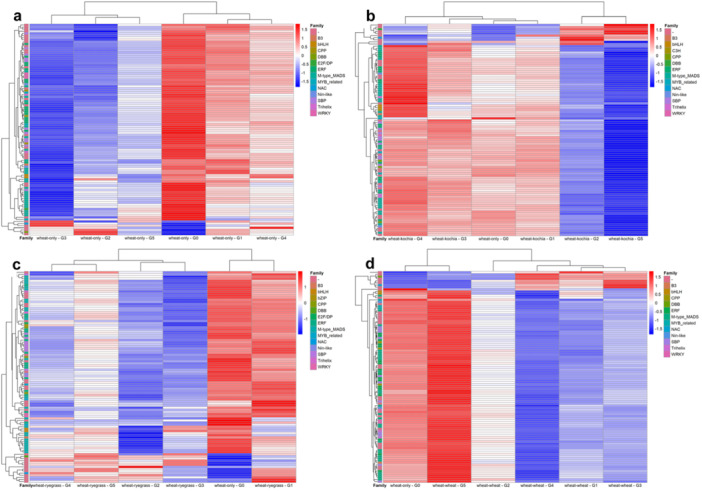
Heatmap of the top 100 most variable genes across generations for the treatments across generations (G1–G5) with the wheat‐only G0 generation included as a reference. (a) Wheat‐only, (b) wheat‐kochia, (c) wheat‐ryegrass, (d) wheat–wheat. Blue indicates downregulation, red indicates upregulation, and white represents neutral or average expression levels. The hierarchical clustering reveals distinct expression patterns across generations (G0–G5). Gene families are annotated on the left, highlighting key regulatory groups. [Color figure can be viewed at wileyonlinelibrary.com]

The hierarchical clustering heatmaps (Figure 6) display expression patterns of the top 100 most variable genes (ranked by variance in FPKM values) across four treatments compared to WO Generation 0, revealing gene expression changes over five generations. In Figure 6a (WO), Generations 0 and 1 show strong upregulation, while Generations 2 and 5 exhibit pronounced downregulation. Generation 4 remains mostly upregulated, indicating a potential rebound or adaptive response. Notable gene families, such as ERF transcription factors, (Myeloblastosis) MYB_related transcription factors, and M‐type_MADS (MCMI Agamous Deficiens Srf) transcription factors, consistently highlight their roles in stress adaptation. In Figure 6b (WK), upregulation is prominent in Generations 3 and 4, followed by downregulation in Generations 2 and 5. Compared to Generation 0, the kochia‐treated groups show significant shifts, reflecting a strong regulatory response to biotic stress. Key families like NAC (Nam, Ataf, and Cuc) transcription factors, ERF, and MYB‐related exhibit dynamic changes, suggesting their involvement in stress mediation. Figure 6c (WR) reveals strong upregulation in Generation 1, similar to Generation 0, while Generation 3 shows downregulation and Generation 4 displays a mix of upregulated and neutral expression. The distinct changes in ryegrass‐treated generations highlight the impact of competitive stress. Gene families such as bHLH (basic Helix‐Loop‐Helix) transcription factors, ERF, and WRKY transcription factors show considerable variability, reflecting their roles in stress response pathways. Figure 6d (WW) shows a predominant trend of downregulation in Generations 1, 3, and 4, likely due to competitive stress effects. Generation 2 remains largely neutral, while Generation 5 exhibits strong upregulation, suggesting an adaptive rebound or stress memory effect. Key regulatory families like M‐type_MADS, MYB_related, ERF, and WRKY show significant transitions from downregulation to pronounced upregulation in Generation 5, indicating enhanced adaptive responses in repeated WW interactions.

### 2.7 Co‐Expression of DEGs Across Generations

**Figure 7:**
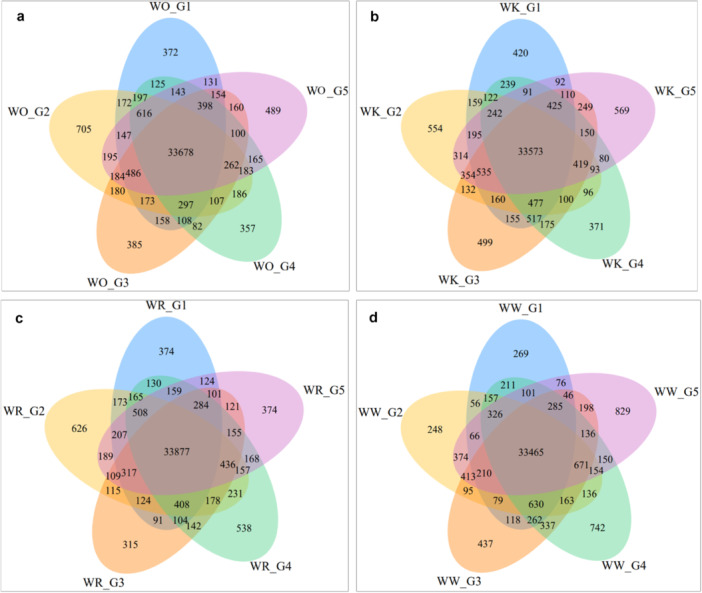
Venn diagram showing the co‐expression of differentially expressed genes across treatment generations. Numbers within each section indicate the unique and shared genes across generations. Overlapping regions show genes co‐expressed between specific generations, while the central region highlights genes consistently co‐expressed across all five generations. (a) WO = wheat‐only, (b) WK = wheat‐kochia, (c) WR = wheat‐ryegrass 3, and (d) WW = wheat–wheat. G = generation. [Color figure can be viewed at wileyonlinelibrary.com]

Venn diagrams illustrating co‐expression patterns of DEGs across generations revealed both unique and shared gene expression responses within each treatment (Figure 7). In the WO treatment, 33 678 genes were consistently co‐expressed across all five generations, with additional generation‐specific subsets observed (Figure 7a). The highest number of unique DEGs was noted in Generation 2 (705), indicating a period of heightened transcriptional change following initial stress exposure. The WK treatment showed a similar pattern, with 33 573 genes co‐expressed across all generations (Figure 7b). However, generation‐specific DEGs were more prominent in Generation 2 (554) and Generation 5 (569), reflecting dynamic transcriptional shifts at both early and late stages of stress adaptation. Generation 3 exhibited substantial overlap with other generations, suggesting stabilisation in gene expression in this generation. For the WR treatment, 33 877 genes were co‐expressed across all five generations, the highest shared gene count among the treatments (Figure 7c). Generation‐specific DEGs were again notable in Generation 2 (626), aligning with the trend of transcriptional reprogramming in response to early competition stress. Generations 3 and 4 demonstrated extensive overlap with other generations, indicating a period of transcriptional equilibrium. In the WW treatment, 33 465 genes were co‐expressed across all generations (Figure 7d). Unique DEGs were most pronounced in Generation 5 (829), suggesting a reactivation of stress‐responsive pathways at the final stage of competition. Similar to other treatments, Generation 2 displayed a relatively high number of unique DEGs (248), emphasising early‐generation transcriptional adjustments.

## 3 Discussion

This study offers new insights into how multigenerational competition stress influences phytohormonal regulation and transcriptomic responses in wheat. By analyzing phytohormonal shifts and DEGs across treatments and generations, we revealed key adaptive and maladaptive responses to prolonged competition from weeds and other wheat plants. These findings build on prior research suggesting that multigenerational stress memory may shape wheat's resource allocation, stress adaptation, and competitive ability (Kwarteng et al. 2025). The observed trends highlight complex interactions between stress signalling pathways, phytohormone regulation, and transcriptional reprogramming, with significant implications for crop resilience and sustainable agriculture.

### 3.1 Integration of Phytohormonal and Transcriptomic Responses With Phenotypic Outcomes

The phytohormonal and transcriptomic changes documented in this study exhibited temporal patterns that corresponded with phenotypic performance observed in our previous work (Kwarteng et al. 2025). In WO treatments, Generation 3 marked a critical transition point by showing a 77% increase in tZ compared to Generation 0, along with peak DEGs (11 729 DEGs), and these molecular changes coincided with maximum biomass and seed production reported previously. The subsequent declines in cytokinins in Generations 4 and 5 and the reduced transcriptomic activity in terms of DEGs aligned with diminished growth performance in later generations, suggesting a shift from adaptive to potentially maladaptive responses. In WK and WR treatments, the relationships between molecular changes and phenotypic outcomes were more complex. Both treatments showed elevated SA in Generations 2 and 3 and progressive increases in DEGs through Generation 5, indicating sustained transcriptional reprogramming under weed competition. However, ABA declined across most generations in these treatments, counter to expectations for resource‐limited conditions. These patterns suggest that the integration of hormonal signals and transcriptional responses does not follow a simple linear relationship with competitive performance.

WW treatment exhibited the highest initial transcriptomic response in terms of DEGs in Generation 1, likely reflecting a rapid detection of neighbour presence, but this response diminished in subsequent generations before resurging in Generation 4. The tZ surge in Generation 5 may suggest a late‐stage reactivation of growth‐promoting pathways, though the functional significance of this response remains unclear. These generation‐specific dynamics illustrate the complex temporal patterning of stress memory, where early, mid, and late generations engage distinct molecular strategies.

### 3.2 Adaptive and Maladaptive Phytohormonal Responses to Multigenerational Competition

Phytohormonal data revealed substantial variation in stress signalling across treatments and generations. SA levels rose significantly in all treatments, especially in Generations 2 and 3, suggesting enhanced SAR and primed pathogen defence (Shah and Zeier 2013). In contrast, JA and JA‐Ile levels declined across treatments, indicating a possible bottleneck in jasmonate‐mediated defences. As SA and JA pathways often act antagonistically, sustained SA elevation could suppress JA responses, potentially weakening herbivore and biotic stress defences (Mur et al. 2013). This trend corresponds with increased OPDA levels, pointing to incomplete activation of the jasmonate pathway under multigenerational stress. The sustained elevation of SA, typically associated with pathogen defence (Verma et al. 2016), alongside suppressed JA responses suggests that wheat may be engaging defence pathways more appropriate for biotic threats rather than the resource competition mechanisms that characterise weed interference.

ABA is a well‐known regulator of plant responses to abiotic stresses, such as drought and salinity, and is also implicated in stress memory (Conrath et al. 2015; Fleta‐Soriano and Munné‐Bosch 2016; Kambona et al. 2023). However, ABA dynamics across generations did not follow patterns expected for resource‐limited conditions. In WO treatments, ABA levels fluctuated without clear directional trends, declining initially before increasing at Generation 3, then declining again in Generations 4 and 5. These fluctuations may reflect the absence of ongoing competitive stress WO in the common garden conditions, where ABA regulation was no longer under selection pressure. In WK and WR treatments, ABA declined persistently across most generations despite these seed lineages originating from plants that experienced competitive stress for resources. This counter‐intuitive pattern suggests that multigenerational exposure to competition may have altered ABA sensitivity or signalling rather than simply maintaining elevated stress hormone levels. The continued suppression of ABA in later generations of weed‐competed lineages may represent a maladaptive legacy effect, where plants have downregulated stress responses that would otherwise aid in resource acquisition, consistent with reports of reduced ABA efficacy under conditions mismatched to the original stress environment (Ma et al. 2018; Schwabe 2025).

Auxins, key regulators of growth and stress responses (Gao et al. 2024), showed increased IAA levels across treatments compared to Generation 0, with the WW treatment exhibiting the highest increase in Generation 4. These increases likely reflect an adaptive response, enhancing root development to improve nutrient acquisition under competition (Tariq and Ahmed 2022). However, a slight reduction in IAA levels in Generation 3 of WK may reflect resource reallocation from growth promotion toward defence or stress regulation pathways. Whether this represents an adaptive stress response or maladaptive growth suppression depends on whether plants would face renewed competition, highlighting the context‐dependency of multigenerational stress memory. Cytokinins, essential for cell division and nutrient allocation (Peleg and Blumwald 2011; Guo et al. 2024), showed mixed responses. tZ increased notably in Generation 3, especially in WW, while cZ and cZR peaked in WK. These changes likely contributed to biomass gains observed in Generation 3 (Kwarteng et al. 2025). However, subsequent cZ and cZR declines in WR and WW may reflect cytokinin reallocation, coinciding with yield and biomass reductions under multigenerational competition (Kwarteng et al. 2025).

### 3.3 Transcriptomic Adaptation to Multigenerational Competition

Transcriptomic profiling revealed significant differences in wheat responses to competition across generations. The WO treatment showed a dynamic progression of gene expression, peaking in DEGs at Generation 3. This surge suggests a heightened transcriptional response driven by stress‐related pathways (Liu et al. 2018). The subsequent decline in DEGs in Generations 4 and 5 reflects a reduced transcriptomic activity compared to the peak observed in Generation 3. This trend may indicate either a stabilisation of the plant's transcriptomic response strategy or a diminished capacity to sustain stress‐responsive gene expression over time (Mayer and Charron 2021). GO analysis showed increased protein synthesis and structural reinforcement, alongside downregulated photosynthesis and carbohydrate transport. These transcriptomic shifts observed despite the absence of competition may reflect multigenerational epigenetic responses from successive generations of selfing rather than competition‐driven adaptations. In maize, inbreeding depression manifests as CHH hypermethylation across transcription factor binding sites, leading to the downregulation of genes involved in chloroplast, mitochondrion, and ribosome functions, independent of environmental stress or competitive interactions (Han et al. 2021).

In WK treatments, the transcriptomic response peaked at Generation 5. Early adaptations included upregulated receptor signalling and nutrient mobilisation; however, reduced photosynthesis in Generation 2 suggests a maladaptive investment in survival mechanisms. Increased ROS detoxification in Generation 3, however, reflects a shift toward adaptive oxidative stress management. The regulation of oxidoreductase activity, both up‐ and downregulated, implies dynamic modulation of ROS signalling, which is crucial for mediating plant responses to abiotic and biotic stress (Mittler et al. 2022). Increased glutathione metabolism and ROS detoxification in Generation 3 signalled a shift towards oxidative stress mitigation (Kumar and Trivedi 2018). This enhanced detoxification capacity at Generation 3 may have contributed to the improved performance observed in Generation 3 wheat under kochia competition. This is consistent with the wheat plant's need to mitigate oxidative damage caused by prolonged kochia competition. Detoxification of ROS is one of the pioneer strategies plants often use in response to stress (Kabir et al. 2021). Concurrently, photosynthetic and polysaccharide metabolic processes remained suppressed, suggesting continued resource reallocation away from energy production and structural reinforcement. By Generation 5, there is a strong upregulation of protein transport processes, particularly Golgi‐associated vesicle membranes and secondary active transmembrane transport, suggesting enhanced intracellular protein trafficking under competitive stress (Zhang et al. 2016). However, lipid and micronutrient transport, including zinc ion transport, were downregulated, potentially signalling a shift in metabolic priorities (Kabir et al. 2021). These changes underscore a dual strategy of stress signalling enhancement and suppressed growth processes to maintain survival under prolonged kochia competition.

The WR treatment showed a moderate yet dynamic transcriptomic response. Early generations prioritised structural reinforcement and metabolic adjustments, with upregulated ion transport, acid phosphatase activity, and transcriptional processes. Generation 2 showed intensified hormonal signalling, including increased oxylipin biosynthesis. By Generation 3, detoxification pathways were upregulated, whereas cell wall metabolism was downregulated, reflecting a shift toward oxidative stress management. Generation 4 emphasised environmental sensing, with increased pattern‐ and polysaccharide‐binding activity, while lipid transport declined. In Generation 5, potassium and zinc ion transport increased, optimising nutrient uptake under prolonged stress, reflecting an adaptive resource acquisition strategy (Ruan et al. 2013).

The WW treatment exhibited distinct transcriptomic shifts across generations, driven by intra‐specific competition. In Generation 1, photosynthesis and polysaccharide metabolism were significantly downregulated, indicating that wheat plants under competitive pressure deprioritized energy production and structural reinforcement in favour of immediate stress responses (Muthusamy and Lee 2024; Upadhyay et al. 2025). Such downregulation may reflect a reallocation of resources to short‐term survival mechanisms, as intra‐specific competition likely limited available resources and space. Ribosomal activity and protein synthesis were upregulated in Generation 2, suggesting an adaptive response to maintain core cellular functions. Increased translational processes could help sustain metabolic and structural repair, despite continued competition (Brostrom and Brostrom 2003; Grant 2011). In Generation 3, upregulated carbohydrate and transmembrane transport activities indicated efforts to improve resource acquisition. This suggests that, under prolonged competition, plants attempt to maximise resource acquisition and utilisation to mitigate ongoing stress (Reich 2018). However, continued suppression of structural and photosynthetic components suggests that plants had not fully mitigated competitive stress effects. Generation 4 showed enhanced environmental sensing and cell wall interactions, likely improving competitive cue detection and resource allocation. Yet, sustained downregulation of thylakoid membranes suggests wheat plants continued prioritising stress tolerance over growth. This sustained downregulation suggests a long‐term adjustment to competitive conditions, where plants may adopt a conservative resource‐use strategy to maintain survival under persistent intra‐specific stress (Freschet et al. 2018).

### 3.4 Phytohormonal Crosstalk and Transcriptional Regulation

Phytohormones, including auxin, cytokinin, gibberellic acid, ABA, JA, ethylene, and SA, act as key second messengers, coordinating stress responses through signal transduction pathways (Salvi et al. 2021). Their interactions with transcription factors contribute to the complexity of stress memory in wheat under multigenerational competition. Transcription factors such as ERF, WRKY, MYB, bZIP, bHLH, and MADS‐box regulate phytohormonal signalling, influencing stress responses, resource allocation, and growth (Aizaz et al. 2024).

In the WO treatment, the sharp increase in SA levels, particularly in Generation 2, likely activated transcription factors such as WRKY, which are known to mediate SAR and stress signalling (Gao et al. 2018). This shift suggests that SA signalling prioritised defence responses at the expense of jasmonate‐driven pathways, as evidenced by a marked reduction in JA and JA‐Ile levels. The decline in JA likely suppressed the activation of MYB‐related transcription factors, which regulate defence and growth trade‐offs (Li et al. 2022), and was accompanied by dynamic changes in bHLH transcription factors, essential regulators of JA‐responsive gene expression (Hickman et al. 2017). However, OPDA accumulation in Generations 2 and 3 suggests continued ROS‐responsive regulation via ERF transcription factors, which mediate stress adaptation and cell wall remodelling (Jisha et al. 2015; Lee et al. 2016; Sun et al. 2017; Zhu et al. 2023). This suggests a partial activation of the jasmonate pathway, with plants possibly relying on OPDA‐mediated defence signalling in the absence of full JA biosynthesis. OPDA, a precursor in the octadecanoid pathway to JA in plants, plays key roles in plant defence and stress responses, and its accumulation may signal a delayed or incomplete jasmonate response (Zhao et al. 2021). For instance, in poplar, loss of the OPDA transporter OPDAT1 resulted in increased OPDA accumulation and reduced JA concentration in plastids (Zhao et al. 2021). Thus, the substantial accumulation of OPDA in almost all treatments and generations, along with reduced JA and JA‐ILE levels compared to Generation 0, may suggest a bottleneck in the conversion of OPDA to JA and JA‐ILE (Christeller and Galis 2014), possibly due to the limited activity of OPDA reductase 3 (OPR3), the enzyme responsible for reducing OPDA to JA (Chini et al. 2018). This OPDA accumulation pattern is particularly relevant to weed competition stress. While complete JA biosynthesis typically responds to herbivore damage and necrotrophic pathogens, OPDA itself can independently regulate stress responses related to oxidative stress and resource limitation (Taki et al. 2005). The sustained OPDA levels without corresponding JA accumulation may reflect a reprogrammed response more suited to the resource‐competitive (abiotic‐like) aspects of weed interference rather than the classical biotic defence pathways that full JA signalling would activate. Fluctuations in ABA levels may have influenced MADS‐box transcription factors involved in metabolic stability and water‐use efficiency (Li et al. 2021). Early cytokinin surges likely activated transcriptional programmes that promote cell division and growth (Peleg and Blumwald 2011), but later declines limited sustained resource allocation, indicating hormonal and transcriptional reprogramming to balance growth and defence.

In WK competition, elevated SA in Generation 2 likely activated WRKY and ERF factors, enhancing defence against kochia's competitive effects. However, suppressed JA and JA‐Ile constrained MYB‐mediated rapid defences. Moderate OPDA accumulation in later generations indicated continued oxidative stress regulation, though to a lesser extent than in WO treatments. Sustained ABA reduction likely weakened resource conservation signals. Cytokinin increases in Generation 3 supported nutrient transport, counterbalancing growth limitations. ERF factors declined across generations, suggesting oxidative stress regulation was secondary, while NAC factors became dominant in Generations 3 and 4, guiding structural adaptation under prolonged stress. The shift from ERF‐mediated oxidative stress responses to NAC‐dominated structural reinforcement suggests that wheat lineages primed with kochia competition prioritised cell wall modification and long‐term architectural changes over rapid stress signalling, consistent with adapting to sustained resource competition rather than acute biotic threats.

WR competition revealed a shift from early defence activation to structural and nutrient management adaptations. WRKY factors upregulated in Generations 1 and 3 correlated with SA peaks, reflecting SAR. JA and JA‐Ile remained suppressed, limiting MYB and bHLH activity. ERF factors, which regulate oxidative stress, were notably downregulated, indicating oxidative stress management was not the primary adaptation. Structural adaptations became more pronounced, with NAC factors upregulated in Generation 4, likely coordinating cell wall reinforcement and long‐term resilience (Nakano et al. 2015). MYB and MADS‐box factors were moderately expressed, supporting secondary metabolism and growth (Abdullah‐Zawawi et al. 2021). NIN‐like factors emerged as key regulators in later generations, particularly in Generations 4 and 5, where they were significantly upregulated. NIN‐like proteins are a group of transcription factors that act as major nitrate sensors, driving the primary nitrate response within the nucleus of plants and playing essential roles in coordinating nitrogen utilisation, plant growth, and responses to nitrogen availability (Sámano et al. 2024). This suggests that wheat under ryegrass competition activated nutrient uptake and assimilation pathways, possibly enhancing nitrogen transport and utilisation to sustain growth despite competitive pressures. This activation of NIN‐like transcription factors highlights a shift toward improving resource acquisition as part of a broader adaptation strategy. Given that Italian ryegrass is a particularly aggressive nitrogen competitor that rapidly depletes soil N (Sørensen 1992), the upregulation of nitrogen sensing and transport pathways represents a targeted adaptation to the specific resource limitation imposed by this weed species. This treatment‐specific molecular response illustrates how multigenerational stress memory can be tailored to the particular competitive strategy of the interfering species.

In WW competition, transcriptional responses balanced early defence activation with later structural and resource acquisition strategies. WRKY upregulation in early generations reflected SA‐driven SAR, but JA and JA‐Ile remained suppressed, limiting jasmonate‐related bHLH activation. ERF activity was moderate but not dominant in oxidative stress regulation. Over time, transcription shifted toward structural adaptation, with MADS‐box factors highly active in Generation 5, promoting reproductive development and structural integrity (Schilling et al. 2018). MYB‐related transcription factors also became prominent in later generations, likely supporting nutrient transport and secondary metabolic adjustments (Li et al. 2022). NIN‐like factors showed significant upregulation in later generations, suggesting that wheat adapted by enhancing nutrient acquisition pathways to sustain growth under prolonged intraspecific competition. This resource‐focused strategy is consistent with the observed cytokinin activity and increased nutrient transport processes in the final stages of growth. Unlike the inter‐specific weed treatments where allelopathy and divergent resource strategies create complex stress environments, WW competition represents pure resource competition among individuals with identical acquisition mechanisms. The dominance of MADS‐box and MYB factors in later generations, coupled with NIN‐like upregulation, suggests that intra‐specific competition memory is encoded primarily through growth regulation and nutrient acquisition pathways rather than defence‐related transcriptional programmes.

Overall, phytohormonal crosstalk was critical in regulating transcription factors across treatments and generations. SA‐driven WRKY and ERF activation prioritised defence, while suppressed JA and ABA signalling limited jasmonate‐ and drought‐related pathways. OPDA played a key role in ERF‐mediated oxidative stress regulation. Cytokinin‐induced transcription initially supported growth, but later declines reflected trade‐offs between resource acquisition and defence. Importantly, the distinct transcription factor profiles across treatments demonstrate that multigenerational stress memory is not a generic ‘stress response’ but rather encodes information about the specific nature of competitive interactions, whether inter‐specific weed competition with its allelopathic and resource‐partitioning challenges, or intra‐specific crowding with its shared resource demands. These findings underscore the complex regulatory mechanisms that balance wheat's adaptive responses to multigenerational competition stress.

### 3.5 Implications for Crop Improvement and Sustainable Agriculture

The transcriptomic and phytohormonal shifts observed in this study advance understanding of the molecular mechanisms underlying multigenerational stress memory in wheat. By identifying key genes, pathways, and hormonal regulators involved in stress memory across generations, this research provides candidate targets for further investigation. However, several critical steps are required before these findings can inform practical breeding strategies. First, the phenotypic consequences of these molecular changes must be validated under field conditions with on‐site weed competition. Our laboratory experiments measured stress memory in the absence of active competition, but whether these molecular signatures translate to enhanced competitive performance when plants encounter weeds again remains untested. Second, generalisations across wheat varieties, environmental conditions, and weed species requires systematic evaluation. Third, the potential trade‐offs between enhanced competitive memory and other agronomic traits such as yield potential, grain quality, and stress tolerance under non‐competitive conditions must be characterised.

The risk of maladaptive responses is particularly important. The decline in performance after Generation 3 in WO treatments, coupled with sustained ABA suppression in weed‐primed lineages, demonstrates that prolonged exposure can lead to detrimental legacy effects. Breeding strategies aimed at enhancing competitive ability through stress priming would need to carefully manage these trade‐offs, potentially targeting the Generation 3 window where adaptive responses appear maximal, or identifying genetic markers that distinguish adaptive from maladaptive trajectories. Ultimately, while this study identifies molecular mechanisms that may contribute to competitive ability, the pathway from mechanistic understanding to herbicide reduction in agriculture requires extensive validation. These findings are best viewed as foundational knowledge that clarifies how wheat integrates and ‘remembers’ competitive stress across generations, rather than as immediate solutions for weed management.

### 3.6 Potential Role of Epigenetic Regulation in Multigenerational Stress Memory

While this study elucidated phytohormonal and transcriptomic mechanisms of multigenerational stress memory, the upstream regulatory mechanisms that transmit these responses across generations remain to be determined. Emerging evidence suggests that epigenetic modifications, including DNA methylation, histone modifications, and small RNA pathways, can stably transmit stress‐responsive states across generations without altering DNA sequences (Crisp et al. 2016; Ramírez‐Carrasco et al. 2017; Kambona et al. 2023). The transcriptomic changes observed, particularly the generation‐specific shifts in transcription factor families (ERF, WRKY, NAC, MYB), may be regulated by heritable chromatin states. In Arabidopsis, stress‐induced histone modifications at transcription factor loci enable enhanced re‐activation of stress‐responsive genes in subsequent exposures (Ding et al. 2012), a mechanism that could explain the primed transcriptional responses we documented. Similarly, DNA methylation patterns can be altered by environmental stress and transmitted through meiosis, potentially encoding information about parental stress history (Crisp et al. 2016).

Of particular relevance to our findings, the WO treatment showed substantial transcriptomic changes despite the absence of competition across generations. Inbreeding depression in maize involves CHH hypermethylation at transcription factor binding sites, leading to downregulation of genes involved in chloroplast, mitochondrion, and ribosome functions independent of environmental stress (Han et al. 2021). A similar mechanism could contribute to the progressive downregulation of photosynthetic and metabolic genes we observed in WO lineages, raising the question of whether our results reflect competition‐induced stress memory, selfing‐induced epigenetic changes, or an interaction between both processes.

Small RNAs, particularly microRNAs and small interfering RNAs, also play roles in transgenerational stress memory by targeting stress‐responsive transcripts and guiding DNA methylation (Hilker et al. 2016; Kambona et al. 2023). The hormone‐responsive transcription factors identified (e.g., MYB, NAC, WRKY families) are known targets of stress‐responsive microRNAs (Reyes and Chua 2007; Fang et al. 2014), suggesting a potential layer of post‐transcriptional regulation that could be heritable.

Importantly, we cannot determine from our data whether the observed stress memory is reinforced by stable epigenetic marks or represents primarily plastic transcriptional responses that might be reversed in subsequent generations. Future studies integrating whole‐genome bisulfite sequencing to map DNA methylation, chromatin immunoprecipitation sequencing (ChIP‐seq) to assess histone modifications, ATAC‐seq to profile chromatin accessibility, and small RNA sequencing would clarify the epigenetic architecture underlying the stress memory documented. Such integrative approaches would also help distinguish true transgenerational epigenetic inheritance from parental effects or carry‐over of maternal transcripts in seeds.

## 4 Methods

### 4.1 Plant Material and Growth Conditions

Greenhouse trials were conducted in 2023 at the Kimberly Research and Extension Center, University of Idaho (42.549877 N, −114.349615 W), with the trial repeated in 2024. The trials used the soft white spring wheat variety ‘UI Cookie’, developed by the University of Idaho's wheat breeding programme at the Aberdeen Research and Extension Center. Released in 2020, ‘UI Cookie’ is known for its excellent processing qualities, high yield potential, and resistance to the Fusarium head scab disease of wheat, barley, and corn. Wheat plants were individually grown in 3‐L pots containing a potting medium (Sunshine Mix LC1, Sun Gro Horticulture Inc., Bellevue, WA) composed of 75% gypsum, perlite, dolomitic limestone, sphagnum peat moss, and a wetting agent. The mix had a nutrient composition of 52.05% organic matter, 50 mg/kg nitrogen, 96.58 mg/kg phosphorus, 255 mg/kg potassium, and 10.75 meq/100 g calcium. No additional fertilisers were used during the trials.

Seeds from Generations 0 through 5 were cultivated under a controlled environment with a light cycle of 14 h light and 10 h dark. Daytime temperatures in the greenhouse were maintained at 25°C ± 2°C, while nighttime temperatures were set at 22°C± 2°C. Environmental conditions were continuously tracked using a real‐time data logger from UbiBot (UbiBot USA, Cypress, TX). Irrigation was administered twice daily using an automatic soaker dripline (DripWorks, Willits, CA) with 6‐inch spacing, delivering approximately 650 mL of water per day during 5‐min cycles.

### 4.2 Experimental Procedures and Study Design

Seeds used in these experiments were sourced from previous multigenerational stress priming studies, as described in (Kwarteng et al. 2025). Generation 0 (G0) represents the original ‘UI Cookie’ seed stock obtained from the University of Idaho breeding programme prior to any competitive stress exposure. In summary, these prior studies comprised four treatments: (1) a single wheat plant grown in a 3‐L pot (WO), (2) a wheat plant surrounded by eight kochia plants (WK), (3) a wheat plant surrounded by eight Italian ryegrass plants (WR), and (4) a wheat plant surrounded by eight other wheat plants (WW). To ensure sufficient seed production for the subsequent generations, the experimental design followed a completely randomised layout with 15 replicates per treatment. At physiological maturity, the central wheat plants in each pot were harvested. Seeds from Generation 1 (G1) plants were used to plant Generation 2 (G2), and this process was repeated for subsequent generations, up to Generation 5 (G3, G4, and G5). To prevent cross‐pollination, polyethylene pollination bags (Seedburo Equipment Co., Des Plaines, IL, USA) were placed over the central wheat plants before anthesis. This sequential process generated wheat seed stocks from five successive generations (G1–G5), each with a distinct history of competition exposure or isolation.

For the phytohormonal and transcriptome profiling experiments reported in the current study, seeds from Generations 0 to 5 across all four treatment lineages were planted individually in 3‐L pots in the summer of 2023 and 2024. The experimental setup was a completely randomised design with four replicates per treatment. Unlike the earlier stress priming treatments in which plants experienced active competition with neighbouring plants, with the exception of WO treatments, these plants were grown in isolation, with a single seed planted in the center of each pot without any surrounding plants, to specifically evaluate heritable changes in phytohormonal profiles and gene expression that persisted independent of active competitive interactions in each generation and treatment.

### 4.3 Sample Collection and Preparation

#### 4.3.1 Leaf Plant Hormone Sample Collection and Preparation

Wheat leaf tissue samples were collected at the fully expanded third leaf stage, as defined by Zadoks’ cereal growth stages scale [#13] (Zadoks et al. 1974). The samples were carefully harvested, rapidly frozen in liquid nitrogen, and stored in a −80°C freezer for 24 h. They were then shipped on dry ice to Creative Proteomics (Creative Proteomics, Shirley, NY) for quantitative analysis. Tissues were homogenised using a mortar and pestle with liquid nitrogen, and quantitative analyses of specific phytohormones were performed using the LC‐MS/MS procedure. An aliquot of 100 mg of tissue was transferred to a tube and the following hormones were extracted: ABA, SA, JA, JA‐Ile, OPDA, IAA, cZ, tZ, cZR, and tZR using 900 μL of cold methanol:acetonitrile (50:50, v/v) spiked with deuterium‐labelled internal standards [mixture of Deuterated Abscisic Acid (D6‐ABA), Deuterated Salicylic Acid (D4‐SA), Deuterated Jasmonic Acid (D2‐JA), Deuterated Indole‐3‐Acetic Acid (D5‐IAA), Deuterated trans‐Zeatin (D5tZ), Deuterated trans‐Zeatin Riboside (D5tZR), Deuterated Gibberellin A1 (D2‐GA1), and Deuterated Gibberellin A12 (D2‐GA12)]. The data was normalised based on internal standards to account for experimental variation and hormone extraction/ionisation efficiency. Briefly, the tissue samples were disrupted using the TissueLyserII (Qiagen) and after centrifugation at 16 000*g*, the supernatants were collected into fresh tubes. The samples were back‐extracted a second time and supernatants were combined before drying down using a speed vacuum.

#### 4.3.2 Leaf Plant Hormone Determination

The pellets were re‐dissolved in 15% methanol and run using an LC‐MS MRM (Multiple Reaction Monitoring) targeted assay. The ZORBAX Eclipse Plus C18 column (2.1 × 100 mm, Agilent) had a flow rate of 0.45 mL/min. The gradient of the mobile phases A (0.1% formic acid) and B (0.1% formic acid/90% acetonitrile) was as follows: 5% B for 1 min to 60% B in 4 min to 100% B in 2 min, held at 100% B for 3 min, to 5% B in 0.5 min. The Shimadzu Nexera 2 LC system was interfaced with a Sciex QTRAP 6500+ mass spectrometer equipped with a TurboIonSpray (TIS) electrospray ion source. The instrument is configured to acquire ions in both negative and positive modes. Analyst software (version 1.6.3) was used to control sample acquisition and data analysis. The hormones were detected using MRM transitions that were optimised using standards. All hormones were quantified using external standard curves created from a series of standard samples having varying quantities of unlabelled hormones and fixed concentrations of the deuterium‐labelled standards mixture. The results were normalised, and the concentrations were reported in ng/g. Hormones not detected in any of the samples were not included in the analysis.

#### 4.3.3 Transcriptome Profiling

Transcriptome profiling of wheat leaf samples was conducted to identify candidate genes involved in long‐term crop‐weed interactions under multigenerational competition. Leaf samples from Generations 0 to 5 (G0–G5) across WO, WK, WR, and WW treatments were analyzed for differential gene expression. Each treatment was compared to Generation 0 WO to track changes in gene expression.

#### 4.3.4 RNA Sample Collection and Preparation

For RNA extraction, wheat leaf tissue samples from seedlings at the fully expanded third leaf stage, as detailed in Zadok's growth scale for cereals [#13] (Zadoks et al. 1974), were collected. The leaves were excised using a pair of sterilised micro‐dissecting laboratory scissors and immediately frozen in liquid nitrogen. The samples were immediately stored in a −80°C freezer for 24 h for the RNA extraction and sequencing procedure using the Novogene platform (Novogene Corporation Inc., Sacramento, CA). Total RNA was extracted using TRIzol reagent (Thermo Scientific, Waltham, MA, USA) and the RNA purity was checked using the NanoPhotometer spectrophotometer (IMPLEN, CA, USA). RNA degradation and contamination was monitored on 1% agarose gels and the integrity and quantitation were assessed using the RNA Nano 6000 Assay Kit of the Bioanalyzer 2100 system (Agilent Technologies, CA, USA). Only RNA with an RNA integrity number (RIN) of > 7.0 was used in the library preparation and sequencing.

#### 4.3.5 cDNA Library Preparation and Illumina Sequencing

A total amount of 1 μg RNA per sample was used as input material for the RNA sample preparations. Sequencing libraries were generated using NEBNext Ultra RNALibrary Prep Kit for Illumina (New England Biolabs, USA) following the manufacturer's recommendations, and index codes were added to attribute sequences to each sample. Briefly, mRNA was purified from total RNA using poly‐T oligo‐attached magnetic beads. Fragmentation was carried out using divalent cations under elevated temperature in NEBNext First Strand Synthesis Reaction Buffer (5X). First‐strand cDNA was synthesised using random hexamer primer and M‐MuLV Reverse Transcriptase (RNase H‐). Second‐strand cDNA synthesis was subsequently performed using DNA Polymerase I and RNase H. Remaining overhangs were converted into blunt ends via exonuclease/polymerase activities. After adenylation of 3′ ends of DNA fragments, NEBNext Adaptors with hairpin loop structure were ligated to prepare for hybridisation. In order to select cDNA fragments of preferentially 150–200 bp in length, the library fragments were purified with the AMPure XP system (Beckman Coulter, Beverly, CA, USA). A total of 3 μL USER Enzyme (New England Biolabs [NEB], Ipswich, MA, USA) was then used with size‐selected, adaptor‐ligated cDNA at 37°C for 15 min followed by 5 min at 95°C before carrying out polymerase chain reaction (PCR). PCR was performed with Phusion High‐Fidelity DNA polymerase, Universal PCR primers, and Index (X) Primer. Finally, PCR products were purified with the AMPure XP system, and library quality was assessed on the Agilent Bioanalyzer 2100 system (Agilent, Santa Clara, CA, USA).

#### 4.3.6 Reference Genome Mapping and Quantification

Raw data (raw reads) of FASTQ format were first processed through fastp. In this step, clean data (clean reads) were obtained by removing reads containing adapter and poly‐N sequences and reads with low quality from raw data. At the same time, Q20, Q30, and GC content of the clean data were calculated. All the downstream analyses were based on clean, high‐quality data. Reference genome (IWGSC RefSeq v2.1) and gene model annotation files were downloaded from the genome website browser (NCBI/UCSC/Ensembl) directly. Paired‐end clean reads were aligned to the reference genome using the Spliced Transcripts Alignment to a Reference (STAR) software, which is based on a previously undescribed RNA‐seq alignment algorithm that uses sequential maximum mappable seed search in uncompressed suffix arrays followed by seed clustering and stitching procedure. STAR exhibits better alignment precision and sensitivity than other RNA‐seq aligners for both experimental and simulated data.

Gene function was annotated using the following databases: COG (Cluster of Orthologous Groups of Proteins), PFAM (Protein Family), GO, KEGG, and KO (KEGG Orthology). Read counts for each mapped gene were generated using FeatureCounts (v1.5.0‐p3), and Reads Per Kilobase of exon model per Million mapped reads (RPKM) of each gene were calculated based on the length of the gene and read counts mapped to this gene. RPKM considers the effect of sequencing depth and gene length for the read counts at the same time and is currently the most commonly used method for estimating gene expression levels (Mortazavi et al. 2008). FPKM values (FPKM > 1) were subsequently calculated based on gene length and read counts. FPKM (expected number of Fragments Per Kilobase of transcript sequence per Millions base pairs sequenced) is the most common method of estimating gene expression levels, which takes the effects into consideration of both sequencing depth and gene length on counting of fragments (Zhao et al. 2020). Pearson correlation analysis was then performed in R, based on the FPKM values.

#### 4.3.7 DEG Analysis and Identification

Differential expression analysis was performed to compare Generation 0 WO with each treatment using the *DESeq2* R package. *DESeq2* provides statistical routines for determining differential expression in digital gene expression data using a model based on the negative binomial distribution. The resulting *p‐*values were adjusted using the Benjamini and Hochberg's approach for controlling the FDR. Genes with an adjusted *p‐*value < 0.05 and an absolute log_2_ (fold change) ≥ 1 (twofold change) found by *DESeq2* were assigned as differentially expressed.

#### 4.3.8 Cluster Analysis, GO Enrichment Analysis of DEGs, and Heatmap

The clustering of the index‐coded samples was performed on a cBot Cluster Generation System using PE Cluster Kit cBot‐HS (Illumina) according to the manufacturer's instructions. After cluster generation, the library preparations were sequenced on an Illumina platform (Nova seq. 6000), and paired‐end reads were generated. GO is the abbreviation of Gene Ontology (http://www.geneontology.org/), which is a major bioinformatics classification system to unify the presentation of gene properties across all species. GO enrichment analysis of DEGs was implemented using the *clusterProfiler* R package (Yu et al. 2012). GO terms with corrected *p‐*value < 0.05 were considered significantly enriched DEGs. Heatmaps were generated using the *pheatmap* package in R (Kolde and Kolde 2015), with hierarchical clustering of FPKM values, using Z‐score normalisation to group genes and samples with similar expression patterns.

#### 4.3.9 Data Collection and Statistical Analysis

All data analyses were carried out in R statistical language version 4.3.1 with the *ggplot, cowplot, dplyr*, and *tidyverse* packages (Wilke 2020; Wickham et al. 2022; R Core Team 2025). To perform the analysis of variance (ANOVA) for each treatment, the dataset was filtered to include only Generation 0 and the specified treatment. A loop was used to assess each variable of interest. Variance checks were performed to ensure that there was an adequate amount of variance for modelling. A linear model was built for each variable using the *lm()* function in the form ‘*variable~gen’*, where generation was treated as a categorical predictor (R Core Team 2025). Pairwise comparisons between Generations 1 and 5 and Generation 0 were performed using the *multcomp* package (R Core Team 2025). Dunnett's test was conducted for each variable using the *glht()* function from the *multcomp* package, which is designed for generalised linear hypotheses. This test was selected as it compares multiple treatment means against a single control (in this case, Generation 0) while controlling for Type I error. To evaluate the relationship between the treatments and each of the tested phytohormones, the *ggplot2* package in R was used to generate forest plots that would allow for the comparison of percentage increase or decrease in treatment responses between Generation 0 and Generations 1–5, while *cowplot* was used to improve the aesthetics and layout.

## Conflicts of Interest

The authors declare no conflicts of interest.

## Supporting information


**Supplementary Figure 1:** Pie charts illustrating the distribution of genome regions across different samples (AK_1, AK_68, AK_84, AKC_15, AKC_37, AKC_61). **Supplementary Figure 2:** Differential expression analysis of wheat‐kochia treatments compared to generation 0 wheat‐only. **Supplementary Figure 3:** Differential expression analysis of wheat‐ryegrass treatments compared to generation 0 wheat‐only. **Supplementary Figure 4:** Differential expression analysis of wheat‐wheat treatments compared to generation 0 wheat‐only. **Supplementary Figure 5:** GO term enrichment analysis of differentially expressed genes for the wheat‐ryegrass treatment. **Supplementary Figure 6:** GO term enrichment analysis of differentially expressed genes for the wheat‐wheat treatment. **Supplementary Table 1:** Sample information for sequencing analysis. Treatments are indicated. **Supplementary Table 2:** Overview of cDNA libraries constructed, showing raw and clean reads across treatments and generations. **Supplementary Table 3:** Read distribution analysis showing alignment statistics for the reference genome. **Supplementary Table 4:** Summary of differential gene expression (*DESeq2*) analysis results for treatment comparisons.

## Data Availability

The data that support the findings of this study are available from the corresponding author (Albert T. Adjesiwor or Albert O. Kwarteng) upon reasonable request.
