# Supplementary material for: Phytohormonal and Transcriptomic Mechanisms of Multigenerational Stress Memory in Wheat Under Weed Competition
Source: Plant Cell Environ. 2026 Mar 11;49(6):3436–54. doi: 10.1111/pce.70475 (PMC13136550; doi:10.1111/pce.70475)
Supplement: Supplementary file 1 — Supplementary Figure 1: Pie charts illustrating the distribution of genome regions across different samples (AK_1, AK_68, AK_84, AKC_15, AKC_37, AKC_61). Supplementary Figure 2: Differential expression analysis of wheat‐kochia treatments compared to generation 0 wheat‐only. Supplementary Figure 3: Differential expression analysis of wheat‐ryegrass treatments compared to generation 0 wheat‐only. Supplementary Figure 4: Differential expression analysis of wheat‐wheat treatments compared to generation 0 wheat‐only. Supplementary Figure 5: GO term enrichment analysis of differentially expressed genes for the wheat‐ryegrass treatment. Supplementary Figure 6: GO term enrichment analysis of differentially expressed genes for the wheat‐wheat treatment. Supplementary Table 1: Sample information for sequencing analysis. Treatments are indicated. Supplementary Table 2: Overview of cDNA libraries constructed, showing raw and clean reads across treatments and generations. Supplementary Table 3: Read distribution analysis showing alignment statistics for the reference genome. Supplementary Table 4: Summary of differential gene expression (DESeq2) analysis results for treatment comparisons. [file PCE-49-3436-s001.DOC]

**Supplementary tables**

## **Supplementary Table 1 |** **Sample information for sequencing analysis**. Treatments are indicated. Wheat_ryegra = wheat-ryegrass, Wheat_only = wheat-only, Wheat_kochia = wheat-kochia; Wheat_ryegra = wheat-ryegrass; Wheat_wheat = wheat-wheat. WO = wheat-only; WK = wheat-kochia; WR = wheat-ryegrass; WW = wheat-wheat. G0 = generation 0; G1 = generation 1; G2 = generation 2; G3 = generation 3; G4 = generation 4; G5 = generation 5.

| **Sample name** | **Species** | | **Treatment** | **Treatment_Generation** |
| --- | --- | --- | --- | --- |
| AKC_1 | | *Triticum aestivum* | Wheat_only | WO_G0 |
| AKC_2 | | *Triticum aestivum* | Wheat_only | WO_G1 |
| AKC_3 | | *Triticum aestivum* | Wheat_wheat | WW_G1 |
| AKC_4 | | *Triticum aestivum* | Wheat_ryegra | WR_G1 |
| AKC_5 | | *Triticum aestivum* | Wheat_kochia | WK_G1 |
| AKC_6 | | *Triticum aestivum* | Wheat_only | WO_G2 |
| AKC_7 | | *Triticum aestivum* | Wheat_wheat | WW_G2 |
| AKC_8 | | *Triticum aestivum* | Wheat_ryegra | WR_G2 |
| AKC_9 | | *Triticum aestivum* | Wheat_kochia | WK_G2 |
| AKC_10 | | *Triticum aestivum* | Wheat_only | WO_G3 |
| AKC_11 | | *Triticum aestivum* | Wheat_wheat | WW_G3 |
| AKC_12 | | *Triticum aestivum* | Wheat_ryegra | WR_G3 |
| AKC_13 | | *Triticum aestivum* | Wheat_kochia | WK_G3 |
| AKC_14 | | *Triticum aestivum* | Wheat_only | WO_G4 |
| AKC_15 | | *Triticum aestivum* | Wheat_wheat | WW_G4 |
| AKC_16 | | *Triticum aestivum* | Wheat_ryegra | WR_G4 |
| AKC_17 | | *Triticum aestivum* | Wheat_kochia | WK_G4 |
| AKC_18 | | *Triticum aestivum* | Wheat_only | WO_G5 |
| AKC_19 | | *Triticum aestivum* | Wheat_wheat | WW_G5 |
| AKC_20 | | *Triticum aestivum* | Wheat_ryegra | WR_G5 |
| AKC_21 | | *Triticum aestivum* | Wheat_kochia | WK_G5 |
| AKC_22 | | *Triticum aestivum* | Wheat_wheat | WW_G1 |
| AKC_23 | | *Triticum aestivum* | Wheat_ryegra | WR_G3 |
| AKC_24 | | *Triticum aestivum* | Wheat_only | WO_G2 |
| AKC_25 | | *Triticum aestivum* | Wheat_wheat | WW_G4 |
| AKC_26 | | *Triticum aestivum* | Wheat_wheat | WW_G3 |
| AKC_27 | | *Triticum aestivum* | Wheat_wheat | WW_G5 |
| AKC_28 | | *Triticum aestivum* | Wheat_kochia | WK_G3 |
| AKC_29 | | *Triticum aestivum* | Wheat_ryegra | WR_G1 |
| AKC_30 | | *Triticum aestivum* | Wheat_only | WO_G1 |
| AKC_31 | | *Triticum aestivum* | Wheat_kochia | WK_G5 |
| AKC_32 | | *Triticum aestivum* | Wheat_only | WO_G5 |
| AKC_33 | | *Triticum aestivum* | Wheat_only | WO_G3 |
| AKC_34 | | *Triticum aestivum* | Wheat_ryegra | WR_G4 |
| AKC_35 | | *Triticum aestivum* | Wheat_ryegra | WR_G5 |
| AKC_36 | | *Triticum aestivum* | Wheat_kochia | WK_G4 |
| AKC_37 | | *Triticum aestivum* | Wheat_wheat | WW_G2 |
| AKC_38 | | *Triticum aestivum* | Wheat_kochia | WK_G1 |
| AKC_39 | | *Triticum aestivum* | Wheat_ryegra | WR_G2 |
| AKC_40 | | *Triticum aestivum* | Wheat_only | WO_G0 |
| AKC_41 | | *Triticum aestivum* | Wheat_only | WO_G4 |
| AKC_42 | | *Triticum aestivum* | Wheat_kochia | WK_G2 |
| AKC_43 | | *Triticum aestivum* | Wheat_ryegra | WR_G1 |
| AKC_44 | | *Triticum aestivum* | Wheat_only | WO_G5 |
| AKC_45 | | *Triticum aestivum* | Wheat_only | WO_G0 |
| AKC_46 | | *Triticum aestivum* | Wheat_kochia | WK_G3 |
| AKC_47 | | *Triticum aestivum* | Wheat_ryegra | WR_G4 |
| AKC_48 | | *Triticum aestivum* | Wheat_kochia | WK_G1 |
| AKC_49 | | *Triticum aestivum* | Wheat_wheat | WW_G4 |
| AKC_50 | | *Triticum aestivum* | Wheat_only | WO_G1 |
| AKC_51 | | *Triticum aestivum* | Wheat_kochia | WK_G4 |
| AKC_52 | | *Triticum aestivum* | Wheat_wheat | WW_G2 |
| AKC_53 | | *Triticum aestivum* | Wheat_wheat | WW_G1 |
| AKC_54 | | *Triticum aestivum* | Wheat_only | WO_G4 |
| AKC_55 | | *Triticum aestivum* | Wheat_wheat | WW_G5 |
| AKC_56 | | *Triticum aestivum* | Wheat_ryegra | WR_G2 |
| AKC_57 | | *Triticum aestivum* | Wheat_kochia | WK_G5 |
| AKC_58 | | *Triticum aestivum* | Wheat_only | WO_G3 |
| AKC_59 | | *Triticum aestivum* | Wheat_ryegra | WR_G5 |
| AKC_60 | | *Triticum aestivum* | Wheat_kochia | WK_G2 |
| AKC_61 | | *Triticum aestivum* | Wheat_only | WO_G2 |
| AKC_62 | | *Triticum aestivum* | Wheat_ryegra | WR_G3 |
| AKC_63 | | *Triticum aestivum* | Wheat_wheat | WW_G3 |
| AKC_64 | | *Triticum aestivum* | Wheat_wheat | WW_G4 |
| AKC_65 | | *Triticum aestivum* | Wheat_kochia | WK_G1 |
| AKC_66 | | *Triticum aestivum* | Wheat_ryegra | WR_G1 |
| AKC_67 | | *Triticum aestivum* | Wheat_ryegra | WR_G4 |
| AKC_68 | | *Triticum aestivum* | Wheat_only | WO_G3 |
| AKC_69 | | *Triticum aestivum* | Wheat_ryegra | WR_G5 |
| AKC_70 | | *Triticum aestivum* | Wheat_wheat | WW_G3 |
| AKC_71 | | *Triticum aestivum* | Wheat_only | WO_G2 |
| AKC_72 | | *Triticum aestivum* | Wheat_wheat | WW_G5 |
| AKC_73 | | *Triticum aestivum* | Wheat_kochia | WK_G4 |
| AKC_75 | | *Triticum aestivum* | Wheat_only | WO_G5 |
| AKC_76 | | *Triticum aestivum* | Wheat_only | WO_G0 |
| AKC_77 | | *Triticum aestivum* | Wheat_wheat | WW_G2 |
| AKC_78 | | *Triticum aestivum* | Wheat_ryegra | WR_G2 |
| AKC_79 | | *Triticum aestivum* | Wheat_kochia | WK_G3 |
| AKC_80 | | *Triticum aestivum* | Wheat_kochia | WK_G5 |
| AKC_81 | | *Triticum aestivum* | Wheat_ryegra | WR_G3 |
| AKC_82 | | *Triticum aestivum* | Wheat_only | WO_G4 |
| AKC_83 | | *Triticum aestivum* | Wheat_only | WO_G1 |
| AKC_84 | | *Triticum aestivum* | Wheat_wheat | WW_G1 |
| AK_1 | | *Triticum aestivum* | Wheat_only | WO_G0 |
| AK_2 | | *Triticum aestivum* | Wheat_only | WO_G1 |
| AK_3 | | *Triticum aestivum* | Wheat_wheat | WW_G1 |
| AK_4 | | *Triticum aestivum* | Wheat_ryegra | WR_G1 |
| AK_5 | | *Triticum aestivum* | Wheat_kochia | WK_G1 |
| AK_6 | | *Triticum aestivum* | Wheat_only | WO_G2 |
| AK_7 | | *Triticum aestivum* | Wheat_wheat | WW_G2 |
| AK_8 | | *Triticum aestivum* | Wheat_ryegra | WR_G2 |
| AK_9 | | *Triticum aestivum* | Wheat_kochia | WK_G2 |
| AK_10 | | *Triticum aestivum* | Wheat_only | WO_G3 |
| AK_11 | | *Triticum aestivum* | Wheat_wheat | WW_G3 |
| AK_12 | | *Triticum aestivum* | Wheat_ryegra | WR_G3 |
| AK_13 | | *Triticum aestivum* | Wheat_kochia | WK_G3 |
| AK_14 | | *Triticum aestivum* | Wheat_only | WO_G4 |
| AK_15 | | *Triticum aestivum* | Wheat_wheat | WW_G4 |
| AK_16 | | *Triticum aestivum* | Wheat_ryegra | WR_G4 |
| AK_17 | | *Triticum aestivum* | Wheat_kochia | WK_G4 |
| AK_18 | | *Triticum aestivum* | Wheat_only | WO_G5 |
| AK_19 | | *Triticum aestivum* | Wheat_wheat | WW_G5 |
| AK_20 | | *Triticum aestivum* | Wheat_ryegra | WR_G5 |
| AK_21 | | *Triticum aestivum* | Wheat_kochia | WK_G5 |
| AK_22 | | *Triticum aestivum* | Wheat_wheat | WW_G1 |
| AK_23 | | *Triticum aestivum* | Wheat_ryegra | WR_G3 |
| AK_24 | | *Triticum aestivum* | Wheat_only | WO_G2 |
| AK_25 | | *Triticum aestivum* | Wheat_wheat | WW_G4 |
| AK_26 | | *Triticum aestivum* | Wheat_wheat | WW_G3 |
| AK_27 | | *Triticum aestivum* | Wheat_wheat | WW_G5 |
| AK_28 | | *Triticum aestivum* | Wheat_kochia | WK_G3 |
| AK_29 | | *Triticum aestivum* | Wheat_ryegra | WR_G1 |
| AK_30 | | *Triticum aestivum* | Wheat_only | WO_G1 |
| AK_31 | | *Triticum aestivum* | Wheat_kochia | WK_G5 |
| AK_32 | | *Triticum aestivum* | Wheat_only | WO_G5 |
| AK_33 | | *Triticum aestivum* | Wheat_only | WO_G3 |
| AK_34 | | *Triticum aestivum* | Wheat_ryegra | WR_G4 |
| AK_35 | | *Triticum aestivum* | Wheat_ryegra | WR_G5 |
| AK_36 | | *Triticum aestivum* | Wheat_kochia | WK_G4 |
| AK_37 | | *Triticum aestivum* | Wheat_wheat | WW_G2 |
| AK_38 | | *Triticum aestivum* | Wheat_kochia | WK_G1 |
| AK_39 | | *Triticum aestivum* | Wheat_ryegra | WR_G2 |
| AK_40 | | *Triticum aestivum* | Wheat_only | WO_G0 |
| AK_41 | | *Triticum aestivum* | Wheat_only | WO_G4 |
| AK_42 | | *Triticum aestivum* | Wheat_kochia | WK_G2 |
| AK_43 | | *Triticum aestivum* | Wheat_ryegra | WR_G1 |
| AK_44 | | *Triticum aestivum* | Wheat_only | WO_G5 |
| AK_45 | | *Triticum aestivum* | Wheat_only | WO_G0 |
| AK_46 | | *Triticum aestivum* | Wheat_kochia | WK_G3 |
| AK_47 | | *Triticum aestivum* | Wheat_ryegra | WR_G4 |
| AK_48 | | *Triticum aestivum* | Wheat_kochia | WK_G1 |
| AK_49 | | *Triticum aestivum* | Wheat_wheat | WW_G4 |
| AK_50 | | *Triticum aestivum* | Wheat_only | WO_G1 |
| AK_51 | | *Triticum aestivum* | Wheat_kochia | WK_G4 |
| AK_52 | | *Triticum aestivum* | Wheat_wheat | WW_G2 |
| AK_53 | | *Triticum aestivum* | Wheat_wheat | WW_G1 |
| AK_54 | | *Triticum aestivum* | Wheat_only | WO_G4 |
| AK_55 | | *Triticum aestivum* | Wheat_wheat | WW_G5 |
| AK_56 | | *Triticum aestivum* | Wheat_ryegra | WR_G2 |
| AK_57 | | *Triticum aestivum* | Wheat_kochia | WK_G5 |
| AK_58 | | *Triticum aestivum* | Wheat_only | WO_G3 |
| AK_59 | | *Triticum aestivum* | Wheat_ryegra | WR_G5 |
| AK_60 | | *Triticum aestivum* | Wheat_kochia | WK_G2 |
| AK_61 | | *Triticum aestivum* | Wheat_only | WO_G2 |
| AK_62 | | *Triticum aestivum* | Wheat_ryegra | WR_G3 |
| AK_63 | | *Triticum aestivum* | Wheat_wheat | WW_G3 |
| AK_64 | | *Triticum aestivum* | Wheat_wheat | WW_G4 |
| AK_65 | | *Triticum aestivum* | Wheat_kochia | WK_G1 |
| AK_66 | | *Triticum aestivum* | Wheat_ryegra | WR_G1 |
| AK_67 | | *Triticum aestivum* | Wheat_ryegra | WR_G4 |
| AK_68 | | *Triticum aestivum* | Wheat_only | WO_G3 |
| AK_69 | | *Triticum aestivum* | Wheat_ryegra | WR_G5 |
| AK_70 | | *Triticum aestivum* | Wheat_wheat | WW_G3 |
| AK_71 | | *Triticum aestivum* | Wheat_only | WO_G2 |
| AK_72 | | *Triticum aestivum* | Wheat_wheat | WW_G5 |
| AK_73 | | *Triticum aestivum* | Wheat_kochia | WK_G4 |
| AK_74 | | *Triticum aestivum* | Wheat_kochia | WK_G2 |
| AK_75 | | *Triticum aestivum* | Wheat_only | WO_G5 |
| AK_76 | | *Triticum aestivum* | Wheat_only | WO_G0 |
| AK_77 | | *Triticum aestivum* | Wheat_wheat | WW_G2 |
| AK_78 | | *Triticum aestivum* | Wheat_ryegra | WR_G2 |
| AK_79 | | *Triticum aestivum* | Wheat_kochia | WK_G3 |
| AK_80 | | *Triticum aestivum* | Wheat_kochia | WK_G5 |
| AK_81 | | *Triticum aestivum* | Wheat_ryegra | WR_G3 |
| AK_82 | | *Triticum aestivum* | Wheat_only | WO_G4 |
| AK_83 | | *Triticum aestivum* | Wheat_only | WO_G1 |
| AK_84 | | *Triticum aestivum* | Wheat_wheat | WW_G1 |

# **Supplementary Table** **2 |** **Overview of cDNA libraries constructed, showing raw and clean reads across treatments and generations**

| sample | library | raw_reads | raw_bases | clean_reads | clean_bases | error_rate | Q20 | Q30 | GC_pct |
| --- | --- | --- | --- | --- | --- | --- | --- | --- | --- |
| AKC_2 | CRAS240009926-2r | 37017636 | 5.55G | 36946266 | 5.54G | 0.01 | 98.81 | 96.38 | 51.82 |
| AKC_3 | CRAS240009927-2r | 41330122 | 6.2G | 41251122 | 6.19G | 0.01 | 98.74 | 96.19 | 53.35 |
| AKC_4 | CRAS240009928-2r | 44145534 | 6.62G | 40292552 | 6.04G | 0.01 | 98.73 | 96.18 | 54.74 |
| AKC_5 | CRAS240009929-2r | 44084016 | 6.61G | 40630746 | 6.09G | 0.01 | 98.71 | 96.09 | 53.79 |
| AKC_6 | CRAS240009930-2r | 40331100 | 6.05G | 40228214 | 6.03G | 0.01 | 98.7 | 96.1 | 54.17 |
| AKC_7 | CRAS240009931-2r | 44741328 | 6.71G | 41094628 | 6.16G | 0.01 | 98.74 | 96.2 | 54.18 |
| AKC_8 | CRAS240009932-2r | 48278094 | 7.24G | 43727440 | 6.56G | 0.01 | 98.7 | 96.06 | 54.12 |
| AKC_9 | CRAS240009933-2r | 55298380 | 8.29G | 50092518 | 7.51G | 0.01 | 98.73 | 96.29 | 52.18 |
| AKC_10 | CRAS240009934-2r | 44744918 | 6.71G | 44662216 | 6.7G | 0.01 | 98.81 | 96.37 | 52.89 |
| AKC_11 | CRAS240009935-2r | 45223018 | 6.78G | 41535846 | 6.23G | 0.01 | 98.78 | 96.29 | 52.8 |
| AKC_12 | CRAS240009936-2r | 38898688 | 5.83G | 38793692 | 5.82G | 0.01 | 98.75 | 96.35 | 51.96 |
| AKC_13 | CRAS240009937-2r | 45643938 | 6.85G | 41801008 | 6.27G | 0.01 | 98.79 | 96.29 | 53.29 |
| AKC_14 | CRAS240009938-2r | 45436266 | 6.82G | 41243950 | 6.19G | 0.01 | 98.8 | 96.35 | 53.18 |
| AKC_15 | CRAS240009939-2r | 42905678 | 6.44G | 39706244 | 5.96G | 0.01 | 98.75 | 96.23 | 53.46 |
| AKC_16 | CRAS240009940-2r | 55393018 | 8.31G | 51643680 | 7.75G | 0.01 | 98.77 | 96.36 | 53.83 |
| AKC_17 | CRAS240009941-2r | 42486982 | 6.37G | 38857338 | 5.83G | 0.01 | 98.81 | 96.38 | 52.72 |
| AKC_18 | CRAS240009942-2r | 45360206 | 6.8G | 41292952 | 6.19G | 0.01 | 98.77 | 96.27 | 54.96 |
| AKC_19 | CRAS240009943-2r | 40203656 | 6.03G | 36618012 | 5.49G | 0.01 | 98.79 | 96.29 | 54.51 |
| AKC_21 | CRAS240009945-2r | 40115536 | 6.02G | 36348014 | 5.45G | 0.01 | 98.8 | 96.34 | 54.19 |
| AKC_22 | CRAS240009946-2r | 44023656 | 6.6G | 39976816 | 6.0G | 0.01 | 98.73 | 96.13 | 53.92 |
| AKC_23 | CRAS240009947-2r | 45581212 | 6.84G | 45504750 | 6.83G | 0.01 | 98.8 | 96.35 | 54.25 |
| AKC_24 | CRAS240009948-2r | 39255202 | 5.89G | 39181684 | 5.88G | 0.01 | 98.78 | 96.33 | 52.47 |
| AKC_25 | CRAS240009949-2r | 43967996 | 6.6G | 43865260 | 6.58G | 0.01 | 98.71 | 96.11 | 52.11 |
| AKC_26 | CRAS240009950-2r | 46020158 | 6.9G | 45926312 | 6.89G | 0.01 | 98.74 | 96.32 | 53.47 |
| AKC_27 | CRAS240009951-2r | 42183114 | 6.33G | 38255906 | 5.74G | 0.01 | 98.83 | 96.41 | 54.42 |
| AKC_28 | CRAS240009952-2r | 38881560 | 5.83G | 38789306 | 5.82G | 0.01 | 98.7 | 96.12 | 54.87 |
| AKC_29 | CRAS240009953-2r | 51217222 | 7.68G | 47202158 | 7.08G | 0.01 | 98.76 | 96.23 | 54.41 |
| AKC_30 | CRAS240009954-2r | 37580576 | 5.64G | 37506416 | 5.63G | 0.01 | 98.77 | 96.29 | 53.49 |
| AKC_31 | CRAS240009955-2r | 51328964 | 7.7G | 46268836 | 6.94G | 0.01 | 98.76 | 96.24 | 54.43 |
| AKC_32 | CRAS240009956-2r | 41715640 | 6.26G | 38449276 | 5.77G | 0.01 | 98.68 | 96.09 | 52.43 |
| AKC_33 | CRAS240009957-2r | 35946198 | 5.39G | 35828676 | 5.37G | 0.01 | 98.48 | 95.36 | 52.8 |
| AKC_34 | CRAS240009958-2r | 37625904 | 5.64G | 37544012 | 5.63G | 0.01 | 98.77 | 96.29 | 53.19 |
| AKC_35 | CRAS240009959-2r | 49891408 | 7.48G | 45228912 | 6.78G | 0.01 | 98.74 | 96.33 | 52.84 |
| AKC_36 | CRAS240009960-1r | 35326738 | 5.3G | 35264888 | 5.29G | 0.01 | 98.78 | 96.34 | 52.62 |
| AKC_37 | CRAS240009961-2r | 41914404 | 6.29G | 41847156 | 6.28G | 0.01 | 98.8 | 96.4 | 52.18 |
| AKC_38 | CRAS240009962-2r | 39832722 | 5.97G | 39760912 | 5.96G | 0.01 | 98.73 | 96.21 | 52.88 |
| AKC_39 | CRAS240009963-2r | 39355196 | 5.9G | 39268296 | 5.89G | 0.01 | 98.68 | 96.13 | 53.18 |
| AKC_40 | CRAS240009964-3r | 43776422 | 6.57G | 43690890 | 6.55G | 0.01 | 98.8 | 96.43 | 52.87 |
| AKC_41 | CRAS240009965-3r | 46193806 | 6.93G | 46116454 | 6.92G | 0.01 | 98.73 | 96.18 | 53.16 |
| AKC_42 | CRAS240009966-3r | 46276250 | 6.94G | 46194174 | 6.93G | 0.01 | 98.77 | 96.29 | 52.12 |
| AKC_43 | CRAS240009967-2r | 54932790 | 8.24G | 54833698 | 8.23G | 0.01 | 98.78 | 96.32 | 53.36 |
| AKC_44 | CRAS240009968-2r | 44472862 | 6.67G | 44375954 | 6.66G | 0.01 | 98.68 | 96.22 | 52.19 |
| AKC_45 | CRAS240009969-2r | 44073762 | 6.61G | 43983386 | 6.6G | 0.01 | 98.74 | 96.26 | 53.11 |
| AKC_46 | CRAS240009970-2r | 46474750 | 6.97G | 43695714 | 6.55G | 0.01 | 98.79 | 96.44 | 54.24 |
| AKC_47 | CRAS240009971-2r | 53012372 | 7.95G | 48186968 | 7.23G | 0.01 | 98.79 | 96.31 | 53.65 |
| AKC_48 | CRAS240009972-2r | 44963658 | 6.74G | 41360012 | 6.2G | 0.01 | 98.77 | 96.25 | 54.05 |
| AKC_49 | CRAS240009973-2r | 40048356 | 6.01G | 39974186 | 6.0G | 0.01 | 98.79 | 96.43 | 52.74 |
| AKC_50 | CRAS240009974-2r | 65420348 | 9.81G | 60334196 | 9.05G | 0.01 | 98.77 | 96.27 | 54.4 |
| AKC_51 | CRAS240009975-2r | 47838046 | 7.18G | 47743386 | 7.16G | 0.01 | 98.74 | 96.2 | 55.85 |
| AKC_52 | CRAS240009976-2r | 44374572 | 6.66G | 41187084 | 6.18G | 0.01 | 98.75 | 96.19 | 54.66 |
| AKC_53 | CRAS240009977-2r | 55447794 | 8.32G | 51224814 | 7.68G | 0.01 | 98.75 | 96.21 | 54.14 |
| AKC_54 | CRAS240009978-1r | 64894010 | 9.73G | 59549308 | 8.93G | 0.01 | 98.85 | 96.45 | 53.52 |
| AKC_55 | CRAS240009979-1r | 48401070 | 7.26G | 44158254 | 6.62G | 0.01 | 98.8 | 96.32 | 53.67 |
| AKC_56 | CRAS240009980-1r | 49137956 | 7.37G | 45889120 | 6.88G | 0.01 | 98.82 | 96.38 | 54.07 |
| AKC_57 | CRAS240009981-1r | 55656810 | 8.35G | 51465956 | 7.72G | 0.01 | 98.75 | 96.19 | 54.67 |
| AKC_58 | CRAS240009982-2r | 44422386 | 6.66G | 41325534 | 6.2G | 0.01 | 98.76 | 96.21 | 54.45 |
| AKC_59 | CRAS240009983-2r | 52978338 | 7.95G | 49202872 | 7.38G | 0.01 | 98.78 | 96.24 | 54.35 |
| AKC_60 | CRAS240009984-2r | 74375732 | 11.16G | 69328876 | 10.4G | 0.01 | 98.79 | 96.3 | 54.51 |
| AKC_61 | CRAS240009985-2r | 52383540 | 7.86G | 47830414 | 7.17G | 0.01 | 98.43 | 95.14 | 53.5 |
| AKC_62 | CRAS240009986-1r | 59161926 | 8.87G | 54999402 | 8.25G | 0.01 | 98.81 | 96.32 | 54.08 |
| AKC_63 | CRAS240009987-2r | 56108166 | 8.42G | 50866246 | 7.63G | 0.01 | 98.84 | 96.44 | 53.3 |
| AKC_64 | CRAS240009988-1r | 78082742 | 11.71G | 71640678 | 10.75G | 0.01 | 98.8 | 96.32 | 53.32 |
| AKC_65 | CRAS240009989-2r | 58160828 | 8.72G | 54088060 | 8.11G | 0.01 | 98.83 | 96.42 | 53.04 |
| AKC_66 | CRAS240009990-2r | 43076352 | 6.46G | 38801160 | 5.82G | 0.01 | 98.78 | 96.42 | 54.09 |
| AKC_67 | CRAS240009991-2r | 43797524 | 6.57G | 39769060 | 5.97G | 0.01 | 98.73 | 96.35 | 53.0 |
| AKC_68 | CRAS240009992-1r | 69922182 | 10.49G | 69793614 | 10.47G | 0.01 | 98.81 | 96.38 | 52.68 |
| AKC_69 | CRAS240009993-2r | 44898602 | 6.73G | 44822142 | 6.72G | 0.01 | 98.82 | 96.37 | 52.47 |
| AKC_70 | CRAS240009994-2r | 47757174 | 7.16G | 47685044 | 7.15G | 0.01 | 98.82 | 96.36 | 52.32 |
| AKC_71 | CRAS240009995-1r | 65483384 | 9.82G | 58978174 | 8.85G | 0.01 | 98.81 | 96.37 | 51.38 |
| AKC_72 | CRAS240009996-2r | 43281950 | 6.49G | 39479722 | 5.92G | 0.01 | 98.84 | 96.45 | 52.95 |
| AKC_73 | CRAS240009997-2r | 53301622 | 8G | 49735208 | 7.46G | 0.01 | 98.87 | 96.48 | 53.73 |
| AKC_75 | CRAS240009999-1r | 53346894 | 8G | 48641384 | 7.3G | 0.01 | 98.76 | 96.26 | 52.11 |
| AKC_76 | CRAS240010000-2r | 50329650 | 7.55G | 50235638 | 7.54G | 0.01 | 98.76 | 96.24 | 51.26 |
| AKC_77 | CRAS240010001-2r | 50947024 | 7.64G | 47434090 | 7.12G | 0.01 | 98.8 | 96.33 | 52.44 |
| AKC_78 | CRAS240010002-2r | 42877942 | 6.43G | 42802752 | 6.42G | 0.01 | 98.76 | 96.25 | 51.97 |
| AKC_79 | CRAS240010003-2r | 45467742 | 6.82G | 45385212 | 6.81G | 0.01 | 98.74 | 96.24 | 51.13 |
| AKC_80 | CRAS240010004-2r | 44325374 | 6.65G | 44250288 | 6.64G | 0.01 | 98.77 | 96.3 | 50.51 |
| AKC_81 | CRAS240010005-2r | 53768502 | 8.07G | 49337644 | 7.4G | 0.01 | 98.74 | 96.16 | 53.38 |
| AKC_82 | CRAS240010006-2r | 90523052 | 13.58G | 82991318 | 12.45G | 0.01 | 98.83 | 96.4 | 52.69 |
| AKC_83 | CRAS240010007-2r | 54303776 | 8.15G | 50044544 | 7.51G | 0.01 | 98.77 | 96.23 | 53.85 |
| AKC_84 | CRAS240010008-1r | 56825238 | 8.52G | 51890678 | 7.78G | 0.01 | 98.76 | 96.22 | 52.91 |
| AK_1 | CRAS240010009-2r | 49018436 | 7.35G | 48922000 | 7.34G | 0.01 | 98.81 | 96.36 | 52.44 |
| AK_2 | CRAS240010010-2r | 53038530 | 7.96G | 49458282 | 7.42G | 0.01 | 98.78 | 96.25 | 54.09 |
| AK_3 | CRAS240010011-2r | 54712604 | 8.21G | 50604676 | 7.59G | 0.01 | 98.81 | 96.38 | 52.48 |
| AK_4 | CRAS240010012-2r | 59126048 | 8.87G | 54282460 | 8.14G | 0.01 | 98.81 | 96.33 | 52.66 |
| AK_5 | CRAS240010013-2r | 52458394 | 7.87G | 48105946 | 7.22G | 0.01 | 98.8 | 96.32 | 53.3 |
| AK_6 | CRAS240010014-2r | 45553446 | 6.83G | 45499974 | 6.82G | 0.01 | 98.75 | 96.35 | 46.5 |
| AK_7 | CRAS240010015-2r | 59328902 | 8.9G | 53778524 | 8.07G | 0.01 | 98.76 | 96.26 | 53.2 |
| AK_8 | CRAS240010016-2r | 58977062 | 8.85G | 53757584 | 8.06G | 0.01 | 98.77 | 96.25 | 51.96 |
| AK_9 | CRAS240010017-2r | 42548688 | 6.38G | 42487824 | 6.37G | 0.01 | 98.79 | 96.45 | 50.29 |
| AK_10 | CRAS240010018-2r | 52209754 | 7.83G | 47424738 | 7.11G | 0.01 | 98.77 | 96.28 | 52.01 |
| AK_11 | CRAS240010019-2r | 51128476 | 7.67G | 46902234 | 7.04G | 0.01 | 98.78 | 96.31 | 52.93 |
| AK_12 | CRAS240010020-2r | 54385340 | 8.16G | 49063798 | 7.36G | 0.01 | 98.81 | 96.38 | 52.72 |
| AK_13 | CRAS240010021-2r | 51025490 | 7.65G | 47309908 | 7.1G | 0.01 | 98.66 | 95.99 | 55.92 |
| AK_14 | CRAS240010022-2r | 43516996 | 6.53G | 39992218 | 6.0G | 0.01 | 98.8 | 96.34 | 51.73 |
| AK_15 | CRAS240010023-2r | 55007450 | 8.25G | 49560582 | 7.43G | 0.01 | 98.75 | 96.27 | 52.49 |
| AK_16 | CRAS240010024-2r | 42981996 | 6.45G | 39189042 | 5.88G | 0.01 | 98.73 | 96.16 | 52.93 |
| AK_17 | CRAS240010025-2r | 52908674 | 7.94G | 48781248 | 7.32G | 0.01 | 98.8 | 96.38 | 53.0 |
| AK_18 | CRAS240010026-2r | 73562570 | 11.03G | 67694082 | 10.15G | 0.01 | 98.89 | 96.55 | 50.82 |
| AK_19 | CRAS240010027-2r | 49233934 | 7.39G | 49137874 | 7.37G | 0.01 | 98.73 | 96.21 | 52.75 |
| AK_20 | CRAS240010028-2r | 45014370 | 6.75G | 44942860 | 6.74G | 0.01 | 98.8 | 96.41 | 51.45 |
| AK_21 | CRAS240010029-2r | 61061604 | 9.16G | 55254740 | 8.29G | 0.01 | 98.78 | 96.34 | 51.23 |
| AK_22 | CRAS240010030-2r | 49794710 | 7.47G | 49709738 | 7.46G | 0.01 | 98.85 | 96.53 | 51.01 |
| AK_23 | CRAS240010031-2r | 62451858 | 9.37G | 58255500 | 8.74G | 0.01 | 98.76 | 96.2 | 53.96 |
| AK_24 | CRAS240010032-2r | 43794192 | 6.57G | 43726020 | 6.56G | 0.01 | 98.82 | 96.43 | 51.89 |
| AK_25 | CRAS240010033-2r | 51172716 | 7.68G | 51061590 | 7.66G | 0.01 | 98.78 | 96.3 | 51.71 |
| AK_26 | CRAS240010034-2r | 49256852 | 7.39G | 44568630 | 6.69G | 0.01 | 98.78 | 96.33 | 52.18 |
| AK_27 | CRAS240010035-2r | 61168598 | 9.18G | 55750416 | 8.36G | 0.01 | 98.79 | 96.34 | 53.22 |
| AK_28 | CRAS240010036-2r | 37648298 | 5.65G | 37576320 | 5.64G | 0.01 | 98.77 | 96.28 | 52.81 |
| AK_29 | CRAS240010037-2r | 65593038 | 9.84G | 60669332 | 9.1G | 0.01 | 98.78 | 96.3 | 52.49 |
| AK_30 | CRAS240010038-1r | 66804268 | 10.02G | 60510202 | 9.08G | 0.01 | 98.85 | 96.5 | 53.05 |
| AK_31 | CRAS240010039-2r | 41757014 | 6.26G | 41680440 | 6.25G | 0.01 | 98.78 | 96.34 | 51.62 |
| AK_32 | CRAS240010040-2r | 76390864 | 11.46G | 76245730 | 11.44G | 0.01 | 98.8 | 96.35 | 52.08 |
| AK_33 | CRAS240010041-2r | 47735754 | 7.16G | 43543356 | 6.53G | 0.01 | 98.86 | 96.49 | 53.55 |
| AK_34 | CRAS240010042-2r | 39938978 | 5.99G | 39864056 | 5.98G | 0.01 | 98.73 | 96.18 | 51.98 |
| AK_35 | CRAS240010043-2r | 54355970 | 8.15G | 54261920 | 8.14G | 0.01 | 98.76 | 96.3 | 50.84 |
| AK_36 | CRAS240010044-2r | 43710138 | 6.56G | 43617630 | 6.54G | 0.01 | 98.7 | 96.14 | 52.3 |
| AK_37 | CRAS240010045-2r | 61337570 | 9.2G | 55332736 | 8.3G | 0.01 | 98.83 | 96.45 | 53.25 |
| AK_38 | CRAS240010046-2r | 50080044 | 7.51G | 45231930 | 6.78G | 0.01 | 98.75 | 96.32 | 53.02 |
| AK_39 | CRAS240010047-2r | 51436492 | 7.72G | 51348536 | 7.7G | 0.01 | 98.77 | 96.32 | 52.49 |
| AK_40 | CRAS240010048-2r | 53099024 | 7.96G | 49099388 | 7.36G | 0.01 | 98.73 | 96.15 | 54.61 |
| AK_41 | CRAS240010049-2r | 108714718 | 16.31G | 108528518 | 16.28G | 0.01 | 98.83 | 96.43 | 54.77 |
| AK_42 | CRAS240010050-2r | 55271744 | 8.29G | 50130476 | 7.52G | 0.01 | 98.8 | 96.35 | 55.16 |
| AK_43 | CRAS240010051-2r | 58803152 | 8.82G | 58685940 | 8.8G | 0.01 | 98.77 | 96.26 | 52.95 |
| AK_44 | CRAS240010052-2r | 67024158 | 10.05G | 66897186 | 10.03G | 0.01 | 98.78 | 96.28 | 53.29 |
| AK_45 | CRAS240010053-2r | 58234336 | 8.74G | 53394350 | 8.01G | 0.01 | 98.77 | 96.28 | 53.96 |
| AK_46 | CRAS240010054-2r | 45654040 | 6.85G | 41786550 | 6.27G | 0.01 | 98.8 | 96.31 | 53.06 |
| AK_47 | CRAS240010055-2r | 61370422 | 9.21G | 56328280 | 8.45G | 0.01 | 98.79 | 96.31 | 51.88 |
| AK_48 | CRAS240010056-2r | 40198738 | 6.03G | 36689696 | 5.5G | 0.01 | 98.76 | 96.24 | 53.24 |
| AK_49 | CRAS240010057-2r | 53554690 | 8.03G | 49070272 | 7.36G | 0.01 | 98.81 | 96.39 | 53.58 |
| AK_50 | CRAS240010058-2r | 61182202 | 9.18G | 61062776 | 9.16G | 0.01 | 98.74 | 96.26 | 53.04 |
| AK_51 | CRAS240010059-2r | 61346766 | 9.2G | 55952782 | 8.39G | 0.01 | 98.73 | 96.13 | 53.13 |
| AK_52 | CRAS240010060-2r | 42095284 | 6.31G | 38457664 | 5.77G | 0.01 | 98.77 | 96.21 | 55.03 |
| AK_53 | CRAS240010061-2r | 60342328 | 9.05G | 55605300 | 8.34G | 0.01 | 98.77 | 96.28 | 54.23 |
| AK_54 | CRAS240010062-2r | 47547902 | 7.13G | 47473718 | 7.12G | 0.01 | 98.85 | 96.5 | 51.74 |
| AK_55 | CRAS240010063-2r | 135510054 | 20.33G | 124103034 | 18.62G | 0.01 | 98.8 | 96.36 | 53.68 |
| AK_56 | CRAS240010064-2r | 58466424 | 8.77G | 53804744 | 8.07G | 0.01 | 98.81 | 96.32 | 54.15 |
| AK_57 | CRAS240010065-2r | 67099382 | 10.06G | 61961258 | 9.29G | 0.01 | 98.81 | 96.33 | 52.89 |
| AK_58 | CRAS240010066-2r | 62677794 | 9.4G | 58144686 | 8.72G | 0.01 | 98.78 | 96.26 | 54.02 |
| AK_59 | CRAS240010067-1r | 48955308 | 7.34G | 48873234 | 7.33G | 0.01 | 98.79 | 96.29 | 53.96 |
| AK_60 | CRAS240010068-1r | 47162506 | 7.07G | 47088270 | 7.06G | 0.01 | 98.84 | 96.48 | 52.4 |
| AK_61 | CRAS240010069-1r | 52219558 | 7.83G | 52132336 | 7.82G | 0.01 | 98.78 | 96.32 | 52.36 |
| AK_62 | CRAS240010070-1r | 49387326 | 7.41G | 49294692 | 7.39G | 0.01 | 98.81 | 96.41 | 52.75 |
| AK_63 | CRAS240010071-1r | 47826486 | 7.17G | 43097516 | 6.46G | 0.01 | 98.74 | 96.19 | 53.24 |
| AK_64 | CRAS240010072-1r | 110851134 | 16.63G | 110649780 | 16.6G | 0.01 | 98.82 | 96.42 | 52.61 |
| AK_65 | CRAS240010073-2r | 53151770 | 7.97G | 53052402 | 7.96G | 0.01 | 98.7 | 96.4 | 52.68 |
| AK_66 | CRAS240010074-1r | 62720230 | 9.41G | 62601556 | 9.39G | 0.01 | 98.78 | 96.31 | 54.15 |
| AK_67 | CRAS240010075-1r | 39376896 | 5.91G | 39308988 | 5.9G | 0.01 | 98.8 | 96.4 | 52.22 |
| AK_68 | CRAS240010076-1r | 45857912 | 6.88G | 45763548 | 6.86G | 0.01 | 98.72 | 96.18 | 52.6 |
| AK_69 | CRAS240010077-1r | 50257914 | 7.54G | 50170266 | 7.53G | 0.01 | 98.79 | 96.32 | 54.37 |
| AK_70 | CRAS240010078-2r | 51887844 | 7.78G | 46740928 | 7.01G | 0.01 | 98.77 | 96.56 | 51.4 |
| AK_71 | CRAS240010079-1r | 46180866 | 6.93G | 46089602 | 6.91G | 0.01 | 98.77 | 96.37 | 51.04 |
| AK_72 | CRAS240010080-3r | 39233260 | 5.88G | 39156876 | 5.87G | 0.01 | 98.65 | 96.42 | 51.01 |
| AK_73 | CRAS240010081-1r | 58355624 | 8.75G | 52821698 | 7.92G | 0.01 | 98.77 | 96.25 | 54.51 |
| AK_74 | CRAS240010082-2r | 39497408 | 5.92G | 39427872 | 5.91G | 0.01 | 98.72 | 96.45 | 52.6 |
| AK_75 | CRAS240010083-1r | 46235910 | 6.94G | 46147528 | 6.92G | 0.01 | 98.74 | 96.19 | 53.05 |
| AK_76 | CRAS240010084-2r | 46359046 | 6.95G | 46260910 | 6.94G | 0.01 | 98.72 | 96.14 | 53.32 |
| AK_77 | CRAS240010085-2r | 52159474 | 7.82G | 52067842 | 7.81G | 0.01 | 98.79 | 96.33 | 53.22 |
| AK_78 | CRAS240010086-2r | 85347342 | 12.8G | 85183898 | 12.78G | 0.01 | 98.79 | 96.35 | 52.92 |
| AK_79 | CRAS240010087-2r | 54936418 | 8.24G | 49621392 | 7.44G | 0.01 | 98.72 | 96.16 | 54.78 |
| AK_80 | CRAS240010088-2r | 51343298 | 7.7G | 51249574 | 7.69G | 0.01 | 98.76 | 96.28 | 53.0 |
| AK_81 | CRAS240010089-2r | 48393162 | 7.26G | 48297988 | 7.24G | 0.01 | 98.75 | 96.28 | 51.6 |
| AK_82 | CRAS240010090-1r | 38036148 | 5.71G | 37967140 | 5.7G | 0.01 | 98.78 | 96.35 | 52.03 |
| AK_83 | CRAS240010091-1r | 49977686 | 7.5G | 49884416 | 7.48G | 0.01 | 98.68 | 96.08 | 54.38 |
| AK_84 | CRAS240010092-1r | 52947624 | 7.94G | 52849550 | 7.93G | 0.01 | 98.76 | 96.3 | 53.08 |

**Supplementary Table 3 |** **Read distribution analysis showing alignment statistics for the reference genome**

| **sample** | **total_reads** | **total_map** | **unique_map** | **multi_map** | **read1_map** | **read2_map** | **positive_map** | **negative_map** | **splice_map** | **unsplice_map** | **proper_map** |
| --- | --- | --- | --- | --- | --- | --- | --- | --- | --- | --- | --- |
| AKC_2 | 36946266 | 32052238(86.75%) | 30559759(82.71%) | 1492479(4.04%) | 15310176(41.44%) | 15249583(41.28%) | 15285048(41.37%) | 15274711(41.34%) | 8552812(23.15%) | 22006947(59.56%) | 27003218(73.09%) |
| AKC_3 | 41251122 | 35844437(86.89%) | 33853221(82.07%) | 1991216(4.83%) | 16962825(41.12%) | 16890396(40.95%) | 16915756(41.01%) | 16937465(41.06%) | 9434486(22.87%) | 24418735(59.2%) | 30264208(73.37%) |
| AKC_4 | 40292552 | 34725215(86.18%) | 32684595(81.12%) | 2040620(5.06%) | 16376949(40.65%) | 16307646(40.47%) | 16331086(40.53%) | 16353509(40.59%) | 9384653(23.29%) | 23299942(57.83%) | 29105356(72.24%) |
| AKC_5 | 40630746 | 34853329(85.78%) | 32986199(81.19%) | 1867130(4.6%) | 16537004(40.7%) | 16449195(40.48%) | 16492583(40.59%) | 16493616(40.59%) | 9538096(23.48%) | 23448103(57.71%) | 28600776(70.39%) |
| AKC_6 | 40228214 | 35294318(87.74%) | 33372118(82.96%) | 1922200(4.78%) | 16723850(41.57%) | 16648268(41.38%) | 16678576(41.46%) | 16693542(41.5%) | 8769933(21.8%) | 24602185(61.16%) | 30277806(75.27%) |
| AKC_7 | 41094628 | 35401031(86.15%) | 33364059(81.19%) | 2036972(4.96%) | 16722135(40.69%) | 16641924(40.5%) | 16675231(40.58%) | 16688828(40.61%) | 9423503(22.93%) | 23940556(58.26%) | 28611900(69.62%) |
| AKC_8 | 43727440 | 37557186(85.89%) | 35330284(80.8%) | 2226902(5.09%) | 17721072(40.53%) | 17609212(40.27%) | 17660952(40.39%) | 17669332(40.41%) | 9827842(22.48%) | 25502442(58.32%) | 30164428(68.98%) |
| AKC_9 | 50092518 | 42811565(85.46%) | 40791513(81.43%) | 2020052(4.03%) | 20441577(40.81%) | 20349936(40.62%) | 20390719(40.71%) | 20400794(40.73%) | 10443613(20.85%) | 30347900(60.58%) | 35603160(71.07%) |
| AKC_10 | 44662216 | 37710383(84.43%) | 35530019(79.55%) | 2180364(4.88%) | 17792326(39.84%) | 17737693(39.72%) | 17756605(39.76%) | 17773414(39.8%) | 9442589(21.14%) | 26087430(58.41%) | 31227328(69.92%) |
| AKC_11 | 41535846 | 35474424(85.41%) | 33795392(81.36%) | 1679032(4.04%) | 16929299(40.76%) | 16866093(40.61%) | 16897782(40.68%) | 16897610(40.68%) | 9162492(22.06%) | 24632900(59.31%) | 29987756(72.2%) |
| AKC_12 | 38793692 | 33012489(85.1%) | 31498926(81.2%) | 1513563(3.9%) | 15787391(40.7%) | 15711535(40.5%) | 15745658(40.59%) | 15753268(40.61%) | 7401907(19.08%) | 24097019(62.12%) | 28055044(72.32%) |
| AKC_13 | 41801008 | 35037955(83.82%) | 32977025(78.89%) | 2060930(4.93%) | 16522213(39.53%) | 16454812(39.36%) | 16479574(39.42%) | 16497451(39.47%) | 9137967(21.86%) | 23839058(57.03%) | 28493040(68.16%) |
| AKC_14 | 41243950 | 35334301(85.67%) | 33546236(81.34%) | 1788065(4.34%) | 16800294(40.73%) | 16745942(40.6%) | 16767774(40.66%) | 16778462(40.68%) | 9224071(22.36%) | 24322165(58.97%) | 29873050(72.43%) |
| AKC_15 | 39706244 | 33182570(83.57%) | 31378380(79.03%) | 1804190(4.54%) | 15728276(39.61%) | 15650104(39.41%) | 15687917(39.51%) | 15690463(39.52%) | 9482897(23.88%) | 21895483(55.14%) | 26142566(65.84%) |
| AKC_16 | 51643680 | 42707580(82.7%) | 40057395(77.56%) | 2650185(5.13%) | 20069979(38.86%) | 19987416(38.7%) | 20015145(38.76%) | 20042250(38.81%) | 12075541(23.38%) | 27981854(54.18%) | 32861646(63.63%) |
| AKC_17 | 38857338 | 32131774(82.69%) | 30441478(78.34%) | 1690296(4.35%) | 15247619(39.24%) | 15193859(39.1%) | 15210717(39.15%) | 15230761(39.2%) | 7689397(19.79%) | 22752081(58.55%) | 26321982(67.74%) |
| AKC_18 | 41292952 | 34062943(82.49%) | 31740836(76.87%) | 2322107(5.62%) | 15906757(38.52%) | 15834079(38.35%) | 15848363(38.38%) | 15892473(38.49%) | 9445394(22.87%) | 22295442(53.99%) | 26539560(64.27%) |
| AKC_19 | 36618012 | 30148349(82.33%) | 28298542(77.28%) | 1849807(5.05%) | 14183333(38.73%) | 14115209(38.55%) | 14137533(38.61%) | 14161009(38.67%) | 7955237(21.72%) | 20343305(55.56%) | 23216384(63.4%) |
| AKC_21 | 36348014 | 29861023(82.15%) | 27889727(76.73%) | 1971296(5.42%) | 13972687(38.44%) | 13917040(38.29%) | 13932260(38.33%) | 13957467(38.4%) | 8351947(22.98%) | 19537780(53.75%) | 23050640(63.42%) |
| AKC_22 | 39976816 | 32836583(82.14%) | 30917288(77.34%) | 1919295(4.8%) | 15493403(38.76%) | 15423885(38.58%) | 15453568(38.66%) | 15463720(38.68%) | 9898617(24.76%) | 21018671(52.58%) | 25615500(64.08%) |
| AKC_23 | 45504750 | 38029407(83.57%) | 35685531(78.42%) | 2343876(5.15%) | 17871845(39.27%) | 17813686(39.15%) | 17832161(39.19%) | 17853370(39.23%) | 9867684(21.68%) | 25817847(56.74%) | 30620878(67.29%) |
| AKC_24 | 39181684 | 32982363(84.18%) | 31360231(80.04%) | 1622132(4.14%) | 15708035(40.09%) | 15652196(39.95%) | 15676960(40.01%) | 15683271(40.03%) | 7765788(19.82%) | 23594443(60.22%) | 27707286(70.71%) |
| AKC_25 | 43865260 | 36972056(84.29%) | 35190675(80.22%) | 1781381(4.06%) | 17631829(40.2%) | 17558846(40.03%) | 17597023(40.12%) | 17593652(40.11%) | 9107747(20.76%) | 26082928(59.46%) | 30174628(68.79%) |
| AKC_26 | 45926312 | 36974638(80.51%) | 34901455(75.99%) | 2073183(4.51%) | 17492867(38.09%) | 17408588(37.91%) | 17436359(37.97%) | 17465096(38.03%) | 8973178(19.54%) | 25928277(56.46%) | 27943380(60.84%) |
| AKC_27 | 38255906 | 31081991(81.25%) | 29032404(75.89%) | 2049587(5.36%) | 14545973(38.02%) | 14486431(37.87%) | 14498640(37.9%) | 14533764(37.99%) | 8138744(21.27%) | 20893660(54.62%) | 23523608(61.49%) |
| AKC_28 | 38789306 | 32894092(84.8%) | 31104463(80.19%) | 1789629(4.61%) | 15589827(40.19%) | 15514636(40.0%) | 15547599(40.08%) | 15556864(40.11%) | 8785274(22.65%) | 22319189(57.54%) | 26841756(69.2%) |
| AKC_29 | 47202158 | 39017228(82.66%) | 36824649(78.01%) | 2192579(4.65%) | 18447368(39.08%) | 18377281(38.93%) | 18415558(39.01%) | 18409091(39.0%) | 12114105(25.66%) | 24710544(52.35%) | 31190624(66.08%) |
| AKC_30 | 37506416 | 31008515(82.68%) | 29236202(77.95%) | 1772313(4.73%) | 14650265(39.06%) | 14585937(38.89%) | 14607522(38.95%) | 14628680(39.0%) | 7419857(19.78%) | 21816345(58.17%) | 24750336(65.99%) |
| AKC_31 | 46268836 | 39416701(85.19%) | 37155497(80.3%) | 2261204(4.89%) | 18610743(40.22%) | 18544754(40.08%) | 18573070(40.14%) | 18582427(40.16%) | 11119364(24.03%) | 26036133(56.27%) | 32711326(70.7%) |
| AKC_32 | 38449276 | 31652439(82.32%) | 29871680(77.69%) | 1780759(4.63%) | 14967061(38.93%) | 14904619(38.76%) | 14932092(38.84%) | 14939588(38.86%) | 7852056(20.42%) | 22019624(57.27%) | 24898314(64.76%) |
| AKC_33 | 35828676 | 29264636(81.68%) | 27686728(77.28%) | 1577908(4.4%) | 13884968(38.75%) | 13801760(38.52%) | 13843411(38.64%) | 13843317(38.64%) | 7535002(21.03%) | 20151726(56.24%) | 23323352(65.1%) |
| AKC_34 | 37544012 | 31321685(83.43%) | 29630738(78.92%) | 1690947(4.5%) | 14850582(39.56%) | 14780156(39.37%) | 14812505(39.45%) | 14818233(39.47%) | 7897450(21.04%) | 21733288(57.89%) | 25184912(67.08%) |
| AKC_35 | 45228912 | 38416100(84.94%) | 36545494(80.8%) | 1870606(4.14%) | 18313801(40.49%) | 18231693(40.31%) | 18267915(40.39%) | 18277579(40.41%) | 9062532(20.04%) | 27482962(60.76%) | 31831176(70.38%) |
| AKC_36 | 35264888 | 29813264(84.54%) | 28155808(79.84%) | 1657456(4.7%) | 14100570(39.98%) | 14055238(39.86%) | 14069879(39.9%) | 14085929(39.94%) | 6969254(19.76%) | 21186554(60.08%) | 25191578(71.44%) |
| AKC_37 | 41847156 | 35565090(84.99%) | 33951574(81.13%) | 1613516(3.86%) | 16997805(40.62%) | 16953769(40.51%) | 16969233(40.55%) | 16982341(40.58%) | 7963063(19.03%) | 25988511(62.1%) | 30812970(73.63%) |
| AKC_38 | 39760912 | 33292479(83.73%) | 31637704(79.57%) | 1654775(4.16%) | 15848168(39.86%) | 15789536(39.71%) | 15808711(39.76%) | 15828993(39.81%) | 7350226(18.49%) | 24287478(61.08%) | 27246886(68.53%) |
| AKC_39 | 39268296 | 32834819(83.62%) | 31197656(79.45%) | 1637163(4.17%) | 15630880(39.81%) | 15566776(39.64%) | 15596311(39.72%) | 15601345(39.73%) | 7481852(19.05%) | 23715804(60.39%) | 27448320(69.9%) |
| AKC_40 | 43690890 | 38558408(88.25%) | 36832019(84.3%) | 1726389(3.95%) | 18445206(42.22%) | 18386813(42.08%) | 18409386(42.14%) | 18422633(42.17%) | 8375847(19.17%) | 28456172(65.13%) | 33747008(77.24%) |
| AKC_41 | 46116454 | 38212033(82.86%) | 36367554(78.86%) | 1844479(4.0%) | 18214787(39.5%) | 18152767(39.36%) | 18181696(39.43%) | 18185858(39.43%) | 8183300(17.74%) | 28184254(61.12%) | 32549264(70.58%) |
| AKC_42 | 46194174 | 37510293(81.2%) | 35605730(77.08%) | 1904563(4.12%) | 17830341(38.6%) | 17775389(38.48%) | 17796723(38.53%) | 17809007(38.55%) | 8818052(19.09%) | 26787678(57.99%) | 30047280(65.05%) |
| AKC_43 | 54833698 | 44229427(80.66%) | 41883098(76.38%) | 2346329(4.28%) | 20984513(38.27%) | 20898585(38.11%) | 20929457(38.17%) | 20953641(38.21%) | 10997693(20.06%) | 30885405(56.33%) | 34735222(63.35%) |
| AKC_44 | 44375954 | 36236399(81.66%) | 34508520(77.76%) | 1727879(3.89%) | 17298694(38.98%) | 17209826(38.78%) | 17244272(38.86%) | 17264248(38.9%) | 7929489(17.87%) | 26579031(59.9%) | 29313972(66.06%) |
| AKC_45 | 43983386 | 36089462(82.05%) | 34230554(77.83%) | 1858908(4.23%) | 17151893(39.0%) | 17078661(38.83%) | 17103689(38.89%) | 17126865(38.94%) | 8620654(19.6%) | 25609900(58.23%) | 28679992(65.21%) |
| AKC_46 | 43695714 | 35862789(82.07%) | 33340807(76.3%) | 2521982(5.77%) | 16707365(38.24%) | 16633442(38.07%) | 16653632(38.11%) | 16687175(38.19%) | 10855912(24.84%) | 22484895(51.46%) | 26900500(61.56%) |
| AKC_47 | 48186968 | 39221703(81.39%) | 37180129(77.16%) | 2041574(4.24%) | 18622165(38.65%) | 18557964(38.51%) | 18589679(38.58%) | 18590450(38.58%) | 10669691(22.14%) | 26510438(55.02%) | 31706338(65.8%) |
| AKC_48 | 41360012 | 33869223(81.89%) | 31845994(77.0%) | 2023229(4.89%) | 15957760(38.58%) | 15888234(38.41%) | 15923819(38.5%) | 15922175(38.5%) | 10707725(25.89%) | 21138269(51.11%) | 26231772(63.42%) |
| AKC_49 | 39974186 | 32587983(81.52%) | 30888777(77.27%) | 1699206(4.25%) | 15475687(38.71%) | 15413090(38.56%) | 15436495(38.62%) | 15452282(38.66%) | 8649573(21.64%) | 22239204(55.63%) | 25690100(64.27%) |
| AKC_50 | 60334196 | 49513956(82.07%) | 46479877(77.04%) | 3034079(5.03%) | 23291593(38.6%) | 23188284(38.43%) | 23230329(38.5%) | 23249548(38.53%) | 15204897(25.2%) | 31274980(51.84%) | 38850430(64.39%) |
| AKC_51 | 47743386 | 35227361(73.78%) | 32573026(68.23%) | 2654335(5.56%) | 16325771(34.19%) | 16247255(34.03%) | 16261185(34.06%) | 16311841(34.17%) | 10203366(21.37%) | 22369660(46.85%) | 24451966(51.22%) |
| AKC_52 | 41187084 | 34053287(82.68%) | 31866722(77.37%) | 2186565(5.31%) | 15967466(38.77%) | 15899256(38.6%) | 15925916(38.67%) | 15940806(38.7%) | 10484608(25.46%) | 21382114(51.91%) | 26089496(63.34%) |
| AKC_53 | 51224814 | 43836752(85.58%) | 41368236(80.76%) | 2468516(4.82%) | 20723366(40.46%) | 20644870(40.3%) | 20677855(40.37%) | 20690381(40.39%) | 14101444(27.53%) | 27266792(53.23%) | 36436904(71.13%) |
| AKC_54 | 59549308 | 51168509(85.93%) | 48287456(81.09%) | 2881053(4.84%) | 24193465(40.63%) | 24093991(40.46%) | 24137390(40.53%) | 24150066(40.55%) | 15497459(26.02%) | 32789997(55.06%) | 41757080(70.12%) |
| AKC_55 | 44158254 | 37831990(85.67%) | 35554573(80.52%) | 2277417(5.16%) | 17814273(40.34%) | 17740300(40.17%) | 17770072(40.24%) | 17784501(40.27%) | 11088501(25.11%) | 24466072(55.41%) | 30236948(68.47%) |
| AKC_56 | 45889120 | 39312075(85.67%) | 36991753(80.61%) | 2320322(5.06%) | 18535862(40.39%) | 18455891(40.22%) | 18495937(40.31%) | 18495816(40.31%) | 12880159(28.07%) | 24111594(52.54%) | 31692078(69.06%) |
| AKC_57 | 51465956 | 44089131(85.67%) | 41429754(80.5%) | 2659377(5.17%) | 20767139(40.35%) | 20662615(40.15%) | 20708180(40.24%) | 20721574(40.26%) | 14162127(27.52%) | 27267627(52.98%) | 35745800(69.46%) |
| AKC_58 | 41325534 | 35564355(86.06%) | 33621699(81.36%) | 1942656(4.7%) | 16852054(40.78%) | 16769645(40.58%) | 16814021(40.69%) | 16807678(40.67%) | 11381640(27.54%) | 22240059(53.82%) | 28789312(69.66%) |
| AKC_59 | 49202872 | 41923429(85.21%) | 39412190(80.1%) | 2511239(5.1%) | 19743197(40.13%) | 19668993(39.98%) | 19698811(40.04%) | 19713379(40.07%) | 12802454(26.02%) | 26609736(54.08%) | 34003276(69.11%) |
| AKC_60 | 69328876 | 59569371(85.92%) | 55750570(80.41%) | 3818801(5.51%) | 27928516(40.28%) | 27822054(40.13%) | 27872058(40.2%) | 27878512(40.21%) | 18532836(26.73%) | 37217734(53.68%) | 48213158(69.54%) |
| AKC_61 | 47830414 | 40125033(83.89%) | 37825671(79.08%) | 2299362(4.81%) | 19000223(39.72%) | 18825448(39.36%) | 18911401(39.54%) | 18914270(39.54%) | 11285550(23.59%) | 26540121(55.49%) | 31805456(66.5%) |
| AKC_62 | 54999402 | 46759213(85.02%) | 43761228(79.57%) | 2997985(5.45%) | 21919630(39.85%) | 21841598(39.71%) | 21869073(39.76%) | 21892155(39.8%) | 13870391(25.22%) | 29890837(54.35%) | 37275804(67.77%) |
| AKC_63 | 50866246 | 43217217(84.96%) | 40710633(80.03%) | 2506584(4.93%) | 20396581(40.1%) | 20314052(39.94%) | 20346148(40.0%) | 20364485(40.04%) | 10872073(21.37%) | 29838560(58.66%) | 35089560(68.98%) |
| AKC_64 | 71640678 | 60573880(84.55%) | 56940994(79.48%) | 3632886(5.07%) | 28525658(39.82%) | 28415336(39.66%) | 28445252(39.71%) | 28495742(39.78%) | 14968834(20.89%) | 41972160(58.59%) | 49195776(68.67%) |
| AKC_65 | 54088060 | 46191478(85.4%) | 43638731(80.68%) | 2552747(4.72%) | 21851824(40.4%) | 21786907(40.28%) | 21813948(40.33%) | 21824783(40.35%) | 13069897(24.16%) | 30568834(56.52%) | 37981370(70.22%) |
| AKC_66 | 38801160 | 33287341(85.79%) | 31386750(80.89%) | 1900591(4.9%) | 15729722(40.54%) | 15657028(40.35%) | 15678765(40.41%) | 15707985(40.48%) | 8505385(21.92%) | 22881365(58.97%) | 28054608(72.3%) |
| AKC_67 | 39769060 | 34325252(86.31%) | 32645056(82.09%) | 1680196(4.22%) | 16362933(41.14%) | 16282123(40.94%) | 16315507(41.03%) | 16329549(41.06%) | 8044175(20.23%) | 24600881(61.86%) | 28639976(72.02%) |
| AKC_68 | 69793614 | 60374872(86.5%) | 57260335(82.04%) | 3114537(4.46%) | 28681037(41.09%) | 28579298(40.95%) | 28629192(41.02%) | 28631143(41.02%) | 17164648(24.59%) | 40095687(57.45%) | 50511394(72.37%) |
| AKC_69 | 44822142 | 39108264(87.25%) | 37213901(83.03%) | 1894363(4.23%) | 18637155(41.58%) | 18576746(41.45%) | 18604047(41.51%) | 18609854(41.52%) | 9781425(21.82%) | 27432476(61.2%) | 32974292(73.57%) |
| AKC_70 | 47685044 | 41017281(86.02%) | 38933510(81.65%) | 2083771(4.37%) | 19496163(40.89%) | 19437347(40.76%) | 19467301(40.82%) | 19466209(40.82%) | 10411035(21.83%) | 28522475(59.81%) | 34001222(71.3%) |
| AKC_71 | 58978174 | 50786765(86.11%) | 48389371(82.05%) | 2397394(4.06%) | 24236447(41.09%) | 24152924(40.95%) | 24201172(41.03%) | 24188199(41.01%) | 13159857(22.31%) | 35229514(59.73%) | 42236702(71.61%) |
| AKC_72 | 39479722 | 33694016(85.35%) | 31883352(80.76%) | 1810664(4.59%) | 15968797(40.45%) | 15914555(40.31%) | 15936290(40.37%) | 15947062(40.39%) | 9737814(24.67%) | 22145538(56.09%) | 28132740(71.26%) |
| AKC_73 | 49735208 | 42147839(84.74%) | 39387136(79.19%) | 2760703(5.55%) | 19729621(39.67%) | 19657515(39.52%) | 19673690(39.56%) | 19713446(39.64%) | 11571518(23.27%) | 27815618(55.93%) | 33622274(67.6%) |
| AKC_75 | 48641384 | 42353909(87.07%) | 40504590(83.27%) | 1849319(3.8%) | 20294647(41.72%) | 20209943(41.55%) | 20254619(41.64%) | 20249971(41.63%) | 10935708(22.48%) | 29568882(60.79%) | 36019048(74.05%) |
| AKC_76 | 50235638 | 42347986(84.3%) | 40337518(80.3%) | 2010468(4.0%) | 20209188(40.23%) | 20128330(40.07%) | 20163686(40.14%) | 20173832(40.16%) | 9231306(18.38%) | 31106212(61.92%) | 34475970(68.63%) |
| AKC_77 | 47434090 | 40304109(84.97%) | 38297455(80.74%) | 2006654(4.23%) | 19193314(40.46%) | 19104141(40.28%) | 19144223(40.36%) | 19153232(40.38%) | 10500862(22.14%) | 27796593(58.6%) | 33014986(69.6%) |
| AKC_78 | 42802752 | 36347776(84.92%) | 34792724(81.29%) | 1555052(3.63%) | 17424617(40.71%) | 17368107(40.58%) | 17390887(40.63%) | 17401837(40.66%) | 8013322(18.72%) | 26779402(62.56%) | 30821476(72.01%) |
| AKC_79 | 45385212 | 39968146(88.06%) | 38475468(84.78%) | 1492678(3.29%) | 19275834(42.47%) | 19199634(42.3%) | 19239015(42.39%) | 19236453(42.38%) | 8682616(19.13%) | 29792852(65.64%) | 34909972(76.92%) |
| AKC_80 | 44250288 | 38526277(87.06%) | 37052544(83.73%) | 1473733(3.33%) | 18557331(41.94%) | 18495213(41.8%) | 18531598(41.88%) | 18520946(41.85%) | 8240058(18.62%) | 28812486(65.11%) | 32925438(74.41%) |
| AKC_81 | 49337644 | 41850738(84.83%) | 39660706(80.39%) | 2190032(4.44%) | 19871495(40.28%) | 19789211(40.11%) | 19831247(40.19%) | 19829459(40.19%) | 12534699(25.41%) | 27126007(54.98%) | 33904196(68.72%) |
| AKC_82 | 82991318 | 70741007(85.24%) | 66955281(80.68%) | 3785726(4.56%) | 33527487(40.4%) | 33427794(40.28%) | 33453144(40.31%) | 33502137(40.37%) | 16916419(20.38%) | 50038862(60.29%) | 58136436(70.05%) |
| AKC_83 | 50044544 | 43257913(86.44%) | 40726096(81.38%) | 2531817(5.06%) | 20405575(40.77%) | 20320521(40.6%) | 20358055(40.68%) | 20368041(40.7%) | 12477287(24.93%) | 28248809(56.45%) | 34954046(69.85%) |
| AKC_84 | 51890678 | 43863532(84.53%) | 41641431(80.25%) | 2222101(4.28%) | 20877897(40.23%) | 20763534(40.01%) | 20822573(40.13%) | 20818858(40.12%) | 12254839(23.62%) | 29386592(56.63%) | 34527934(66.54%) |
| AK_1 | 48922000 | 41041848(83.89%) | 38695863(79.1%) | 2345985(4.8%) | 19388528(39.63%) | 19307335(39.47%) | 19336055(39.52%) | 19359808(39.57%) | 10234845(20.92%) | 28461018(58.18%) | 32151172(65.72%) |
| AK_2 | 49458282 | 41833781(84.58%) | 39147553(79.15%) | 2686228(5.43%) | 19622920(39.68%) | 19524633(39.48%) | 19569041(39.57%) | 19578512(39.59%) | 13485853(27.27%) | 25661700(51.89%) | 32679490(66.07%) |
| AK_3 | 50604676 | 42745540(84.47%) | 40464080(79.96%) | 2281460(4.51%) | 20272943(40.06%) | 20191137(39.9%) | 20228048(39.97%) | 20236032(39.99%) | 12248103(24.2%) | 28215977(55.76%) | 34928894(69.02%) |
| AK_4 | 54282460 | 45499349(83.82%) | 42933969(79.09%) | 2565380(4.73%) | 21502993(39.61%) | 21430976(39.48%) | 21463265(39.54%) | 21470704(39.55%) | 10770025(19.84%) | 32163944(59.25%) | 36189676(66.67%) |
| AK_5 | 48105946 | 40434108(84.05%) | 38051556(79.1%) | 2382552(4.95%) | 19060561(39.62%) | 18990995(39.48%) | 19011917(39.52%) | 19039639(39.58%) | 10181491(21.16%) | 27870065(57.93%) | 32308460(67.16%) |
| AK_6 | 45499974 | 41380995(90.95%) | 39283160(86.34%) | 2097835(4.61%) | 19646458(43.18%) | 19636702(43.16%) | 19631241(43.15%) | 19651919(43.19%) | 10011565(22.0%) | 29271595(64.33%) | 36528592(80.28%) |
| AK_7 | 53778524 | 47045415(87.48%) | 44914624(83.52%) | 2130791(3.96%) | 22507668(41.85%) | 22406956(41.67%) | 22454090(41.75%) | 22460534(41.76%) | 13189086(24.52%) | 31725538(58.99%) | 39804012(74.01%) |
| AK_8 | 53757584 | 46332340(86.19%) | 44168199(82.16%) | 2164141(4.03%) | 22135758(41.18%) | 22032441(40.98%) | 22080674(41.07%) | 22087525(41.09%) | 12481051(23.22%) | 31687148(58.94%) | 38724592(72.04%) |
| AK_9 | 42487824 | 37662762(88.64%) | 35978333(84.68%) | 1684429(3.96%) | 18010482(42.39%) | 17967851(42.29%) | 17984486(42.33%) | 17993847(42.35%) | 7522004(17.7%) | 28456329(66.98%) | 34178480(80.44%) |
| AK_10 | 47424738 | 40161405(84.68%) | 38290286(80.74%) | 1871119(3.95%) | 19188407(40.46%) | 19101879(40.28%) | 19141569(40.36%) | 19148717(40.38%) | 10416439(21.96%) | 27873847(58.77%) | 34007756(71.71%) |
| AK_11 | 46902234 | 40732715(86.85%) | 38588900(82.28%) | 2143815(4.57%) | 19337022(41.23%) | 19251878(41.05%) | 19284711(41.12%) | 19304189(41.16%) | 10902359(23.24%) | 27686541(59.03%) | 34698324(73.98%) |
| AK_12 | 49063798 | 42505211(86.63%) | 40172747(81.88%) | 2332464(4.75%) | 20124959(41.02%) | 20047788(40.86%) | 20076838(40.92%) | 20095909(40.96%) | 11960797(24.38%) | 28211950(57.5%) | 36473938(74.34%) |
| AK_13 | 47309908 | 39950195(84.44%) | 37186996(78.6%) | 2763199(5.84%) | 18658210(39.44%) | 18528786(39.16%) | 18587157(39.29%) | 18599839(39.31%) | 13070871(27.63%) | 24116125(50.97%) | 31013724(65.55%) |
| AK_14 | 39992218 | 33908599(84.79%) | 32277009(80.71%) | 1631590(4.08%) | 16170159(40.43%) | 16106850(40.27%) | 16141420(40.36%) | 16135589(40.35%) | 8991020(22.48%) | 23285989(58.23%) | 28080776(70.22%) |
| AK_15 | 49560582 | 43889203(88.56%) | 42073034(84.89%) | 1816169(3.66%) | 21081938(42.54%) | 20991096(42.35%) | 21035150(42.44%) | 21037884(42.45%) | 10769501(21.73%) | 31303533(63.16%) | 38673600(78.03%) |
| AK_16 | 39189042 | 34274458(87.46%) | 32493664(82.92%) | 1780794(4.54%) | 16285971(41.56%) | 16207693(41.36%) | 16239767(41.44%) | 16253897(41.48%) | 8951142(22.84%) | 23542522(60.07%) | 29352830(74.9%) |
| AK_17 | 48781248 | 42299187(86.71%) | 40012377(82.02%) | 2286810(4.69%) | 20049273(41.1%) | 19963104(40.92%) | 19999599(41.0%) | 20012778(41.03%) | 10305438(21.13%) | 29706939(60.9%) | 35860578(73.51%) |
| AK_18 | 67694082 | 54911131(81.12%) | 52047872(76.89%) | 2863259(4.23%) | 26073346(38.52%) | 25974526(38.37%) | 25997367(38.4%) | 26050505(38.48%) | 11329529(16.74%) | 40718343(60.15%) | 42340698(62.55%) |
| AK_19 | 49137874 | 42880017(87.26%) | 40861521(83.16%) | 2018496(4.11%) | 20478025(41.67%) | 20383496(41.48%) | 20424123(41.56%) | 20437398(41.59%) | 8849328(18.01%) | 32012193(65.15%) | 36529938(74.34%) |
| AK_20 | 44942860 | 39707742(88.35%) | 37991984(84.53%) | 1715758(3.82%) | 19026527(42.33%) | 18965457(42.2%) | 18991603(42.26%) | 19000381(42.28%) | 8600070(19.14%) | 29391914(65.4%) | 35385408(78.73%) |
| AK_21 | 55254740 | 47666817(86.27%) | 45617273(82.56%) | 2049544(3.71%) | 22852452(41.36%) | 22764821(41.2%) | 22799675(41.26%) | 22817598(41.3%) | 9658771(17.48%) | 35958502(65.08%) | 41011958(74.22%) |
| AK_22 | 49709738 | 42454498(85.4%) | 40254759(80.98%) | 2199739(4.43%) | 20159152(40.55%) | 20095607(40.43%) | 20112556(40.46%) | 20142203(40.52%) | 9218581(18.54%) | 31036178(62.43%) | 36665474(73.76%) |
| AK_23 | 58255500 | 50008781(85.84%) | 46978858(80.64%) | 3029923(5.2%) | 23553832(40.43%) | 23425026(40.21%) | 23484881(40.31%) | 23493977(40.33%) | 15823071(27.16%) | 31155787(53.48%) | 40206266(69.02%) |
| AK_24 | 43726020 | 37530710(85.83%) | 35725876(81.7%) | 1804834(4.13%) | 17891280(40.92%) | 17834596(40.79%) | 17853830(40.83%) | 17872046(40.87%) | 8753444(20.02%) | 26972432(61.69%) | 31931044(73.03%) |
| AK_25 | 51061590 | 43892533(85.96%) | 41863567(81.99%) | 2028966(3.97%) | 20983888(41.1%) | 20879679(40.89%) | 20936605(41.0%) | 20926962(40.98%) | 11509100(22.54%) | 30354467(59.45%) | 35836634(70.18%) |
| AK_26 | 44568630 | 38219800(85.75%) | 36376368(81.62%) | 1843432(4.14%) | 18233961(40.91%) | 18142407(40.71%) | 18187306(40.81%) | 18189062(40.81%) | 10600016(23.78%) | 25776352(57.84%) | 31670310(71.06%) |
| AK_27 | 55750416 | 47388309(85.0%) | 44576548(79.96%) | 2811761(5.04%) | 22341242(40.07%) | 22235306(39.88%) | 22274995(39.95%) | 22301553(40.0%) | 12014632(21.55%) | 32561916(58.41%) | 38115212(68.37%) |
| AK_28 | 37576320 | 32653810(86.9%) | 30928031(82.31%) | 1725779(4.59%) | 15497781(41.24%) | 15430250(41.06%) | 15465947(41.16%) | 15462084(41.15%) | 9246057(24.61%) | 21681974(57.7%) | 27532002(73.27%) |
| AK_29 | 60669332 | 52419257(86.4%) | 49979034(82.38%) | 2440223(4.02%) | 25042688(41.28%) | 24936346(41.1%) | 24988809(41.19%) | 24990225(41.19%) | 14267196(23.52%) | 35711838(58.86%) | 43998358(72.52%) |
| AK_30 | 60510202 | 51534583(85.17%) | 48625120(80.36%) | 2909463(4.81%) | 24357584(40.25%) | 24267536(40.1%) | 24293982(40.15%) | 24331138(40.21%) | 14022637(23.17%) | 34602483(57.18%) | 42701718(70.57%) |
| AK_31 | 41680440 | 35594023(85.4%) | 33960493(81.48%) | 1633530(3.92%) | 17014629(40.82%) | 16945864(40.66%) | 16970219(40.72%) | 16990274(40.76%) | 8079870(19.39%) | 25880623(62.09%) | 29978672(71.93%) |
| AK_32 | 76245730 | 64281915(84.31%) | 60874914(79.84%) | 3407001(4.47%) | 30499995(40.0%) | 30374919(39.84%) | 30421868(39.9%) | 30453046(39.94%) | 16452408(21.58%) | 44422506(58.26%) | 50655194(66.44%) |
| AK_33 | 43543356 | 36335631(83.45%) | 34146196(78.42%) | 2189435(5.03%) | 17110172(39.29%) | 17036024(39.12%) | 17059377(39.18%) | 17086819(39.24%) | 10094332(23.18%) | 24051864(55.24%) | 29025138(66.66%) |
| AK_34 | 39864056 | 33545084(84.15%) | 31944852(80.13%) | 1600232(4.01%) | 16006525(40.15%) | 15938327(39.98%) | 15961595(40.04%) | 15983257(40.09%) | 7066201(17.73%) | 24878651(62.41%) | 27472042(68.91%) |
| AK_35 | 54261920 | 46226833(85.19%) | 44313981(81.67%) | 1912852(3.53%) | 22200553(40.91%) | 22113428(40.75%) | 22149599(40.82%) | 22164382(40.85%) | 10017885(18.46%) | 34296096(63.2%) | 39588556(72.96%) |
| AK_36 | 43617630 | 37502105(85.98%) | 35692964(81.83%) | 1809141(4.15%) | 17888885(41.01%) | 17804079(40.82%) | 17840303(40.9%) | 17852661(40.93%) | 7989474(18.32%) | 27703490(63.51%) | 31477938(72.17%) |
| AK_37 | 55332736 | 46278848(83.64%) | 43601010(78.8%) | 2677838(4.84%) | 21844568(39.48%) | 21756442(39.32%) | 21785066(39.37%) | 21815944(39.43%) | 13082537(23.64%) | 30518473(55.15%) | 38007292(68.69%) |
| AK_38 | 45231930 | 38265079(84.6%) | 36231175(80.1%) | 2033904(4.5%) | 18156243(40.14%) | 18074932(39.96%) | 18108558(40.03%) | 18122617(40.07%) | 10030550(22.18%) | 26200625(57.93%) | 31161782(68.89%) |
| AK_39 | 51348536 | 43808270(85.32%) | 41360999(80.55%) | 2447271(4.77%) | 20718791(40.35%) | 20642208(40.2%) | 20673246(40.26%) | 20687753(40.29%) | 10911156(21.25%) | 30449843(59.3%) | 36404426(70.9%) |
| AK_40 | 49099388 | 40769860(83.04%) | 37873907(77.14%) | 2895953(5.9%) | 18987781(38.67%) | 18886126(38.47%) | 18920314(38.53%) | 18953593(38.6%) | 11958437(24.36%) | 25915470(52.78%) | 31706152(64.58%) |
| AK_41 | 1.09E+08 | 91479691(84.29%) | 85058607(78.37%) | 6421084(5.92%) | 42613228(39.26%) | 42445379(39.11%) | 42493427(39.15%) | 42565180(39.22%) | 26593885(24.5%) | 58464722(53.87%) | 73968114(68.16%) |
| AK_42 | 50130476 | 43028372(85.83%) | 40063184(79.92%) | 2965188(5.91%) | 20071572(40.04%) | 19991612(39.88%) | 20017184(39.93%) | 20046000(39.99%) | 12661010(25.26%) | 27402174(54.66%) | 35169992(70.16%) |
| AK_43 | 58685940 | 50487157(86.03%) | 47539810(81.01%) | 2947347(5.02%) | 23825590(40.6%) | 23714220(40.41%) | 23765198(40.5%) | 23774612(40.51%) | 14896039(25.38%) | 32643771(55.62%) | 41565284(70.83%) |
| AK_44 | 66897186 | 56945453(85.12%) | 53532362(80.02%) | 3413091(5.1%) | 26823131(40.1%) | 26709231(39.93%) | 26767487(40.01%) | 26764875(40.01%) | 18122582(27.09%) | 35409780(52.93%) | 46361272(69.3%) |
| AK_45 | 53394350 | 44940367(84.17%) | 42049672(78.75%) | 2890695(5.41%) | 21078424(39.48%) | 20971248(39.28%) | 21029149(39.38%) | 21020523(39.37%) | 15031124(28.15%) | 27018548(50.6%) | 35462614(66.42%) |
| AK_46 | 41786550 | 34934986(83.6%) | 32664519(78.17%) | 2270467(5.43%) | 16377221(39.19%) | 16287298(38.98%) | 16322058(39.06%) | 16342461(39.11%) | 9664163(23.13%) | 23000356(55.04%) | 27172038(65.03%) |
| AK_47 | 56328280 | 48431495(85.98%) | 45815076(81.34%) | 2616419(4.64%) | 22955144(40.75%) | 22859932(40.58%) | 22908603(40.67%) | 22906473(40.67%) | 13425551(23.83%) | 32389525(57.5%) | 40716432(72.28%) |
| AK_48 | 36689696 | 31034431(84.59%) | 29166926(79.5%) | 1867505(5.09%) | 14622352(39.85%) | 14544574(39.64%) | 14580829(39.74%) | 14586097(39.76%) | 9010216(24.56%) | 20156710(54.94%) | 24638214(67.15%) |
| AK_49 | 49070272 | 41242346(84.05%) | 38749874(78.97%) | 2492472(5.08%) | 19410431(39.56%) | 19339443(39.41%) | 19368245(39.47%) | 19381629(39.5%) | 13302479(27.11%) | 25447395(51.86%) | 34125350(69.54%) |
| AK_50 | 61062776 | 49591421(81.21%) | 46475829(76.11%) | 3115592(5.1%) | 23287080(38.14%) | 23188749(37.98%) | 23231131(38.04%) | 23244698(38.07%) | 13669071(22.39%) | 32806758(53.73%) | 38881396(63.67%) |
| AK_51 | 55952782 | 46568543(83.23%) | 43643690(78.0%) | 2924853(5.23%) | 21882822(39.11%) | 21760868(38.89%) | 21824451(39.01%) | 21819239(39.0%) | 15268133(27.29%) | 28375557(50.71%) | 36134622(64.58%) |
| AK_52 | 38457664 | 32438380(84.35%) | 30234212(78.62%) | 2204168(5.73%) | 15154164(39.4%) | 15080048(39.21%) | 15113285(39.3%) | 15120927(39.32%) | 10598397(27.56%) | 19635815(51.06%) | 25353254(65.93%) |
| AK_53 | 55605300 | 46884916(84.32%) | 43906799(78.96%) | 2978117(5.36%) | 22005626(39.57%) | 21901173(39.39%) | 21941254(39.46%) | 21965545(39.5%) | 13980483(25.14%) | 29926316(53.82%) | 38046404(68.42%) |
| AK_54 | 47473718 | 40756997(85.85%) | 38885441(81.91%) | 1871556(3.94%) | 19478794(41.03%) | 19406647(40.88%) | 19434067(40.94%) | 19451374(40.97%) | 10123764(21.32%) | 28761677(60.58%) | 34491382(72.65%) |
| AK_55 | 1.24E+08 | 105813375(85.26%) | 99679936(80.32%) | 6133439(4.94%) | 49948192(40.25%) | 49731744(40.07%) | 49816266(40.14%) | 49863670(40.18%) | 32479607(26.17%) | 67200329(54.15%) | 87047978(70.14%) |
| AK_56 | 53804744 | 45533299(84.63%) | 42668485(79.3%) | 2864814(5.32%) | 21383033(39.74%) | 21285452(39.56%) | 21330991(39.65%) | 21337494(39.66%) | 15183880(28.22%) | 27484605(51.08%) | 35707616(66.37%) |
| AK_57 | 61961258 | 52373071(84.53%) | 49434100(79.78%) | 2938971(4.74%) | 24777944(39.99%) | 24656156(39.79%) | 24714526(39.89%) | 24719574(39.9%) | 16312427(26.33%) | 33121673(53.46%) | 42684670(68.89%) |
| AK_58 | 58144686 | 49801716(85.65%) | 47088781(80.99%) | 2712935(4.67%) | 23607739(40.6%) | 23481042(40.38%) | 23547526(40.5%) | 23541255(40.49%) | 15447701(26.57%) | 31641080(54.42%) | 40821628(70.21%) |
| AK_59 | 48873234 | 41951796(85.84%) | 39571468(80.97%) | 2380328(4.87%) | 19827602(40.57%) | 19743866(40.4%) | 19771669(40.46%) | 19799799(40.51%) | 11584067(23.7%) | 27987401(57.27%) | 35253550(72.13%) |
| AK_60 | 47088270 | 41577905(88.3%) | 39751302(84.42%) | 1826603(3.88%) | 19904585(42.27%) | 19846717(42.15%) | 19864027(42.18%) | 19887275(42.23%) | 10048544(21.34%) | 29702758(63.08%) | 36523318(77.56%) |
| AK_61 | 52132336 | 45818724(87.89%) | 44035553(84.47%) | 1783171(3.42%) | 22058013(42.31%) | 21977540(42.16%) | 22012544(42.22%) | 22023009(42.24%) | 10437091(20.02%) | 33598462(64.45%) | 40081432(76.88%) |
| AK_62 | 49294692 | 42527294(86.27%) | 40529995(82.22%) | 1997299(4.05%) | 20306854(41.19%) | 20223141(41.02%) | 20250138(41.08%) | 20279857(41.14%) | 9580350(19.43%) | 30949645(62.78%) | 35897978(72.82%) |
| AK_63 | 43097516 | 36894581(85.61%) | 35328605(81.97%) | 1565976(3.63%) | 17703976(41.08%) | 17624629(40.89%) | 17661459(40.98%) | 17667146(40.99%) | 9087313(21.09%) | 26241292(60.89%) | 31155358(72.29%) |
| AK_64 | 1.11E+08 | 94028482(84.98%) | 89379456(80.78%) | 4649026(4.2%) | 44799579(40.49%) | 44579877(40.29%) | 44678254(40.38%) | 44701202(40.4%) | 25202627(22.78%) | 64176829(58.0%) | 77855976(70.36%) |
| AK_65 | 53052402 | 44843886(84.53%) | 42481865(80.08%) | 2362021(4.45%) | 21283125(40.12%) | 21198740(39.96%) | 21221935(40.0%) | 21259930(40.07%) | 9615907(18.13%) | 32865958(61.95%) | 37366440(70.43%) |
| AK_66 | 62601556 | 53021247(84.7%) | 49827727(79.6%) | 3193520(5.1%) | 24968119(39.88%) | 24859608(39.71%) | 24904441(39.78%) | 24923286(39.81%) | 16238329(25.94%) | 33589398(53.66%) | 43494184(69.48%) |
| AK_67 | 39308988 | 33799785(85.98%) | 32098735(81.66%) | 1701050(4.33%) | 16081307(40.91%) | 16017428(40.75%) | 16035965(40.79%) | 16062770(40.86%) | 7591163(19.31%) | 24507572(62.35%) | 28732228(73.09%) |
| AK_68 | 45763548 | 38952929(85.12%) | 36952639(80.75%) | 2000290(4.37%) | 18522729(40.47%) | 18429910(40.27%) | 18470818(40.36%) | 18481821(40.39%) | 10193253(22.27%) | 26759386(58.47%) | 32004874(69.94%) |
| AK_69 | 50170266 | 41975872(83.67%) | 39208865(78.15%) | 2767007(5.52%) | 19645355(39.16%) | 19563510(38.99%) | 19581954(39.03%) | 19626911(39.12%) | 10778493(21.48%) | 28430372(56.67%) | 33594886(66.96%) |
| AK_70 | 46740928 | 38631659(82.65%) | 36728495(78.58%) | 1903164(4.07%) | 18404771(39.38%) | 18323724(39.2%) | 18359817(39.28%) | 18368678(39.3%) | 10627963(22.74%) | 26100532(55.84%) | 31428758(67.24%) |
| AK_71 | 46089602 | 38888100(84.37%) | 37205045(80.72%) | 1683055(3.65%) | 18641304(40.45%) | 18563741(40.28%) | 18598733(40.35%) | 18606312(40.37%) | 8695292(18.87%) | 28509753(61.86%) | 32899640(71.38%) |
| AK_72 | 39156876 | 33886016(86.54%) | 32360642(82.64%) | 1525374(3.9%) | 16209845(41.4%) | 16150797(41.25%) | 16163439(41.28%) | 16197203(41.36%) | 6915229(17.66%) | 25445413(64.98%) | 29499292(75.34%) |
| AK_73 | 52821698 | 46871953(88.74%) | 44506520(84.26%) | 2365433(4.48%) | 22297049(42.21%) | 22209471(42.05%) | 22242118(42.11%) | 22264402(42.15%) | 12504979(23.67%) | 32001541(60.58%) | 40634926(76.93%) |
| AK_74 | 39427872 | 33405358(84.73%) | 31852001(80.79%) | 1553357(3.94%) | 15954645(40.47%) | 15897356(40.32%) | 15913361(40.36%) | 15938640(40.42%) | 7535303(19.11%) | 24316698(61.67%) | 27934458(70.85%) |
| AK_75 | 46147528 | 40948158(88.73%) | 39023411(84.56%) | 1924747(4.17%) | 19556496(42.38%) | 19466915(42.18%) | 19510621(42.28%) | 19512790(42.28%) | 11494263(24.91%) | 27529148(59.65%) | 35542380(77.02%) |
| AK_76 | 46260910 | 40895622(88.4%) | 38841228(83.96%) | 2054394(4.44%) | 19465519(42.08%) | 19375709(41.88%) | 19408379(41.95%) | 19432849(42.01%) | 9833373(21.26%) | 29007855(62.7%) | 35302064(76.31%) |
| AK_77 | 52067842 | 45186187(86.78%) | 42632275(81.88%) | 2553912(4.9%) | 21357966(41.02%) | 21274309(40.86%) | 21298162(40.9%) | 21334113(40.97%) | 11377938(21.85%) | 31254337(60.03%) | 38066530(73.11%) |
| AK_78 | 85183898 | 73503933(86.29%) | 69266292(81.31%) | 4237641(4.97%) | 34709695(40.75%) | 34556597(40.57%) | 34605189(40.62%) | 34661103(40.69%) | 19409434(22.79%) | 49856858(58.53%) | 60723244(71.28%) |
| AK_79 | 49621392 | 43355799(87.37%) | 40848387(82.32%) | 2507412(5.05%) | 20474741(41.26%) | 20373646(41.06%) | 20408736(41.13%) | 20439651(41.19%) | 11159759(22.49%) | 29688628(59.83%) | 35930828(72.41%) |
| AK_80 | 51249574 | 45775020(89.32%) | 43500018(84.88%) | 2275002(4.44%) | 21790051(42.52%) | 21709967(42.36%) | 21740549(42.42%) | 21759469(42.46%) | 11942802(23.3%) | 31557216(61.58%) | 40180666(78.4%) |
| AK_81 | 48297988 | 42888828(88.8%) | 41125756(85.15%) | 1763072(3.65%) | 20602800(42.66%) | 20522956(42.49%) | 20545184(42.54%) | 20580572(42.61%) | 9436738(19.54%) | 31689018(65.61%) | 37849118(78.37%) |
| AK_82 | 37967140 | 33408776(87.99%) | 31841150(83.87%) | 1567626(4.13%) | 15952195(42.02%) | 15888955(41.85%) | 15911303(41.91%) | 15929847(41.96%) | 7294588(19.21%) | 24546562(64.65%) | 29070600(76.57%) |
| AK_83 | 49884416 | 43681618(87.57%) | 41534260(83.26%) | 2147358(4.3%) | 20809702(41.72%) | 20724558(41.55%) | 20754976(41.61%) | 20779284(41.65%) | 10051331(20.15%) | 31482929(63.11%) | 37981466(76.14%) |
| AK_84 | 52849550 | 47444207(89.77%) | 45436624(85.97%) | 2007583(3.8%) | 22763089(43.07%) | 22673535(42.9%) | 22705387(42.96%) | 22731237(43.01%) | 11737280(22.21%) | 33699344(63.76%) | 42118050(79.69%) |

**Supplementary Table 4 | Summary of differential gene expression (*DESeq2*) analysis results for treatment comparisons**. up = upregulated genes; down = downregulated genes; all = combination of up and downregulated genes

**treatment generation all up down threshold**

Wheat-only 1 4670 2135 2535 DESeq2 pvalue<=0.05 |log2FoldChange|>=0.0

Wheat-only 2 4580 2233 2347 DESeq2 pvalue<=0.05 |log2FoldChange|>=0.0

Wheat-only 3 11729 4700 7029 DESeq2 pvalue<=0.05

|log2FoldChange|>=0.0

Wheat-only 4 2763 1538 1225 DESeq2 pvalue<=0.05

|log2FoldChange|>=0.0

Wheat-only 5 2130 1313 817 DESeq2 pvalue<=0.05

|log2FoldChange|>=0.0

Wheat-kochia 1 1485 937 548 DESeq2 pvalue<=0.05

|log2FoldChange|>=0.0

Wheat-kochia 2 4555 1921 2634 DESeq2 pvalue<=0.05

|log2FoldChange|>=0.0

Wheat-kochia 3 2247 1597 650 DESeq2 pvalue<=0.05

|log2FoldChange|>=0.0

Wheat-kochia 4 1172 593 579 DESeq2 pvalue<=0.05

|log2FoldChange|>=0.0

Wheat-kochia 5 7045 3281 3764 DESeq2 pvalue<=0.05

|log2FoldChange|>=0.0

Wheat-ryegrass 1 2113 1149 964 DESeq2 pvalue<=0.05

|log2FoldChange|>=0.0

Wheat-ryegrass 2 2994 1828 1166 DESeq2 pvalue<=0.05

|log2FoldChange|>=0.0

Wheat-ryegrass 3 3775 1466 2309 DESeq2 pvalue<=0.05

|log2FoldChange|>=0.0

Wheat-ryegrass 4 2167 1119 1048 DESeq2 pvalue<=0.05

|log2FoldChange|>=0.0

Wheat-ryegrass 5 2566 1552 1014 DESeq2 pvalue<=0.05

|log2FoldChange|>=0.0

Wheat-wheat 1 9849 3564 6285 DESeq2 pvalue<=0.05

|log2FoldChange|>=0.0

Wheat-wheat 2 2860 1560 1300 DESeq2 pvalue<=0.05

|log2FoldChange|>=0.0

Wheat-wheat 3 4050 2326 174 DESeq2 pvalue<=0.05

|log2FoldChange|>=0.0

Wheat-wheat 4 7143 3644 3499 DESeq2 pvalue<=0.05

|log2FoldChange|>=0.0

Wheat-wheat 5 1962 1055 907 DESeq2 pvalue<=0.05 |log2FoldChange|>=0.0

**Supplementary figures**

# **Supplementary Fig. 1 |** **Pie charts illustrating the distribution of genome regions across different samples (AK_1, AK_68, AK_84, AKC_15, AKC_37, AKC_61)**. The proportion of exonic, intronic, and intergenic regions is shown for each sample. Exonic regions dominate in all samples, constituting over 80% of the genome, followed by smaller fractions of intergenic (12-15%) and intronic (2-3%) regions. Variations in intergenic and intronic proportions reflect differences in genome organization among the samples.


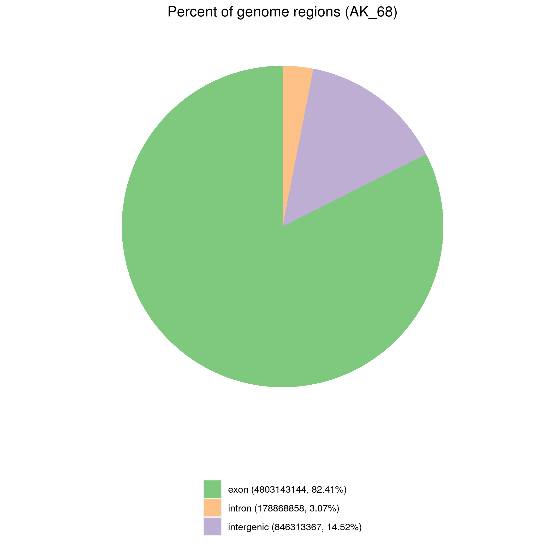

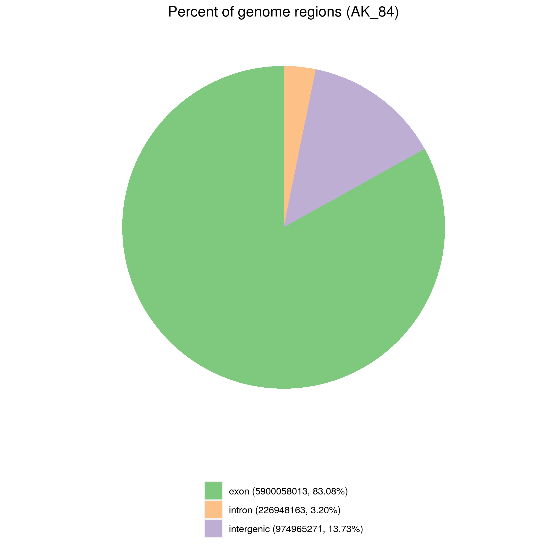

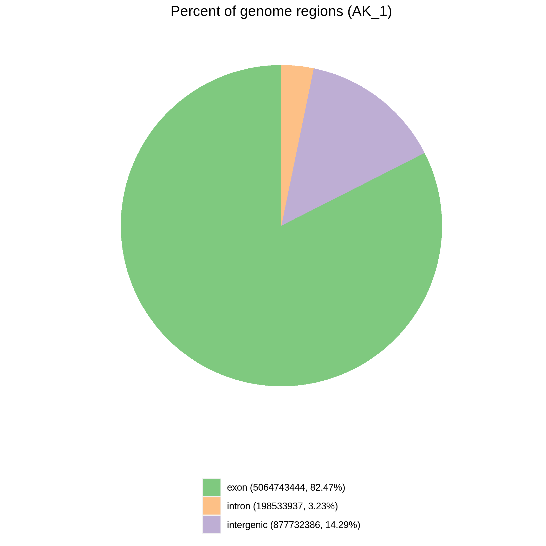

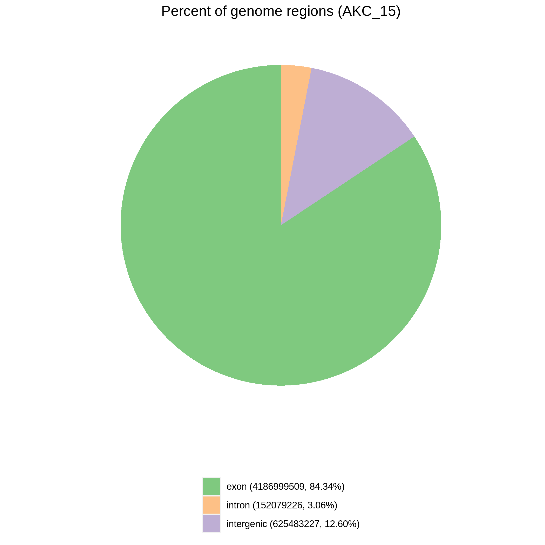

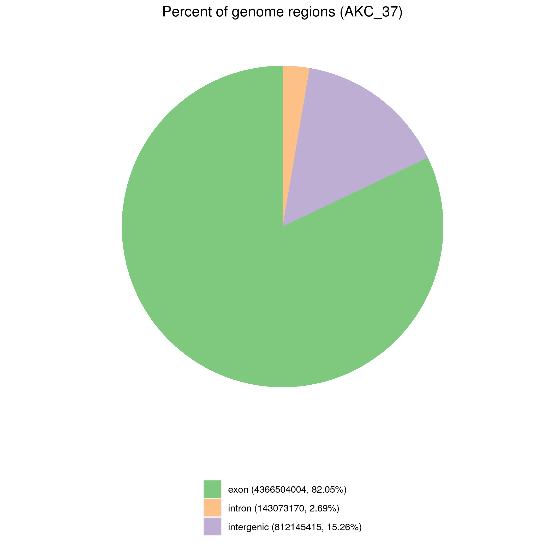

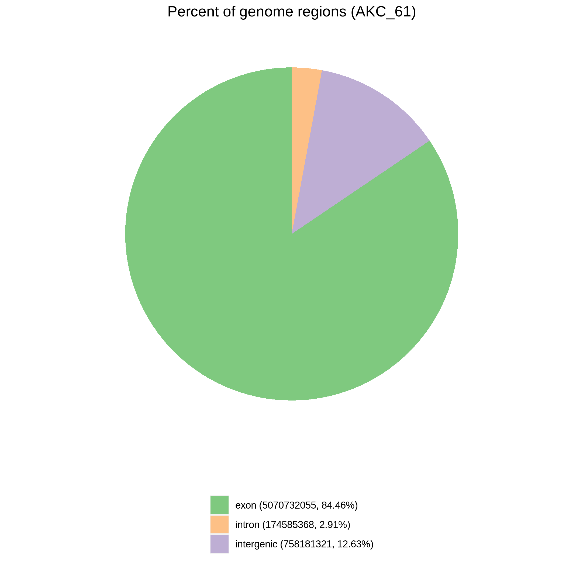


**cN**

**bN**

**aN**

**
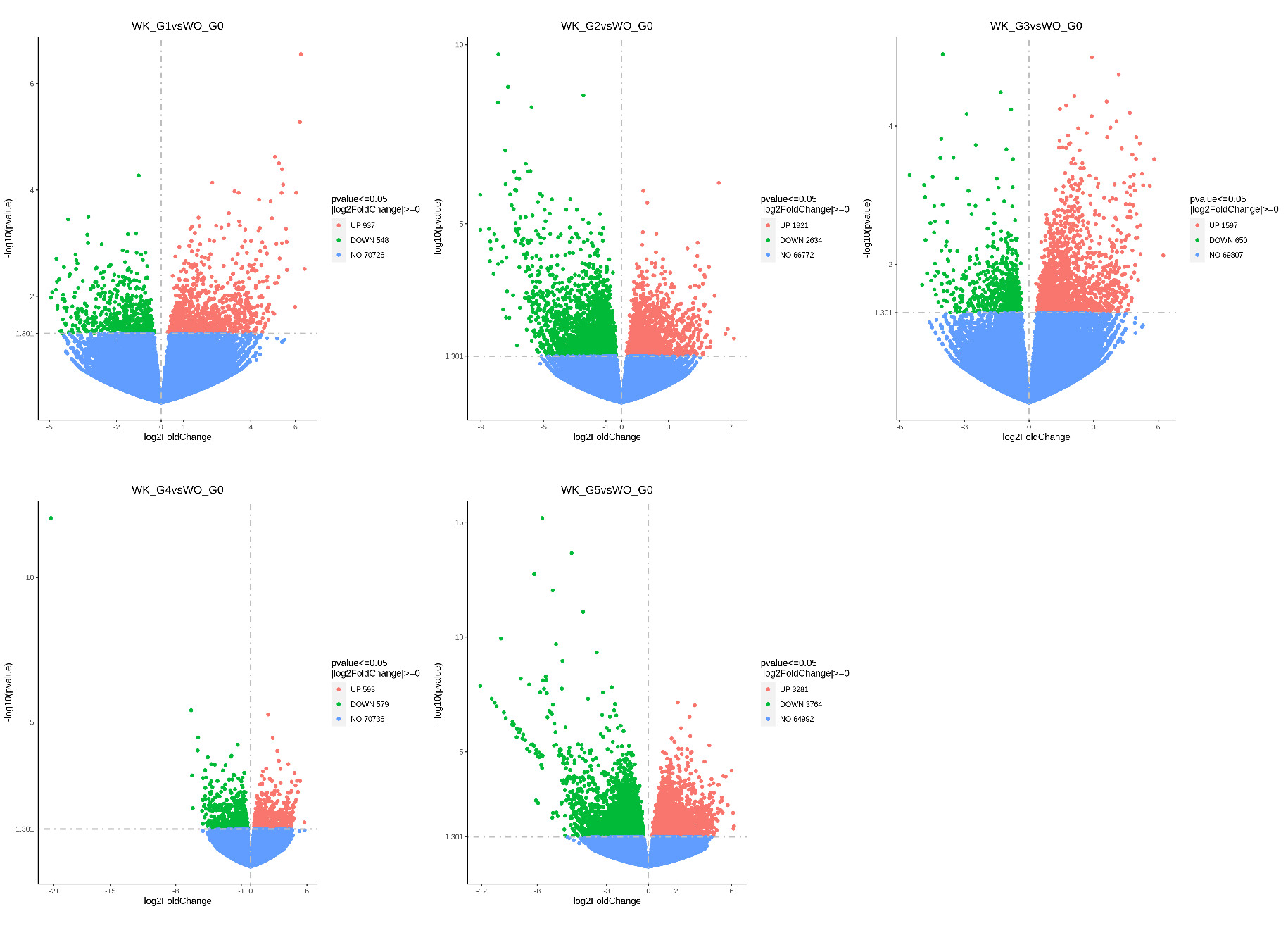
**

**eN**

**d**

**Supplementary Fig. 2 | Differential expression analysis of wheat-kochia treatments compared to generation 0 wheat-only**. Volcano plots illustrate upregulated (red) and downregulated (green) genes (p ≤ 0.05, |log2FoldChange| ≥ 1) relative to wheat-only control conditions. (a) generation 1, (b) generation 2, (c) generation 3, (d) generation 4, and (e) generation 5. Non-significant genes are shown in blue. The number of upregulated, downregulated, and non-significant genes is indicated in the plot legends.

**aN**

**
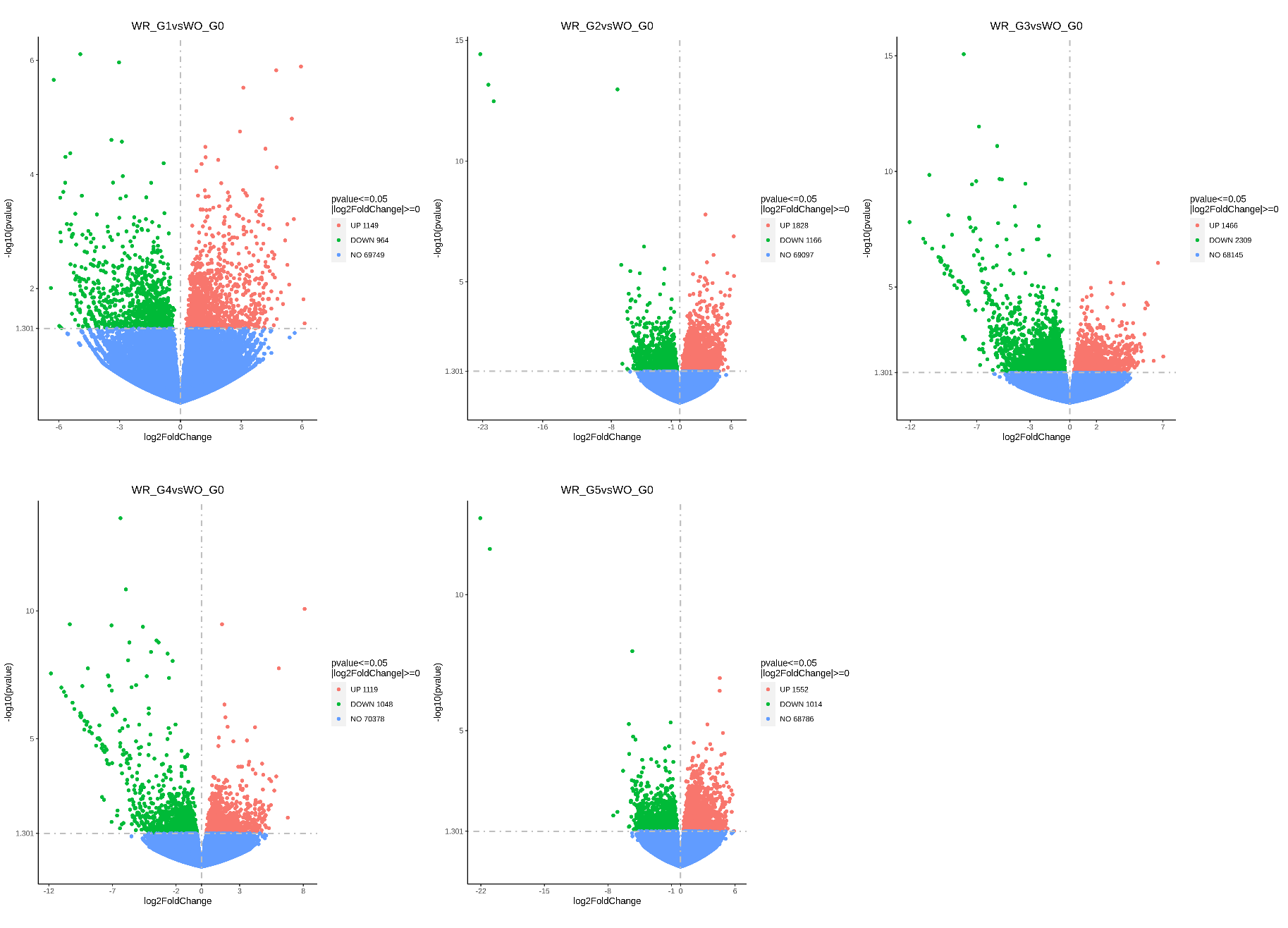
**

**eN**

**dD**

**c**

**b**

**Supplementary Fig. 3 | Differential expression analysis of wheat-ryegrass treatments compared to generation 0 wheat-only**. Volcano plots illustrate upregulated (red) and downregulated (green) genes (p ≤ 0.05, |log2FoldChange| ≥ 1) relative to wheat-only control conditions. (a) generation 1, (b) generation 2, (c) generation 3, (d) generation 4, and (e) generation 5. Non-significant genes are shown in blue. The number of upregulated, downregulated, and non-significant genes is indicated in the plot legends.

**
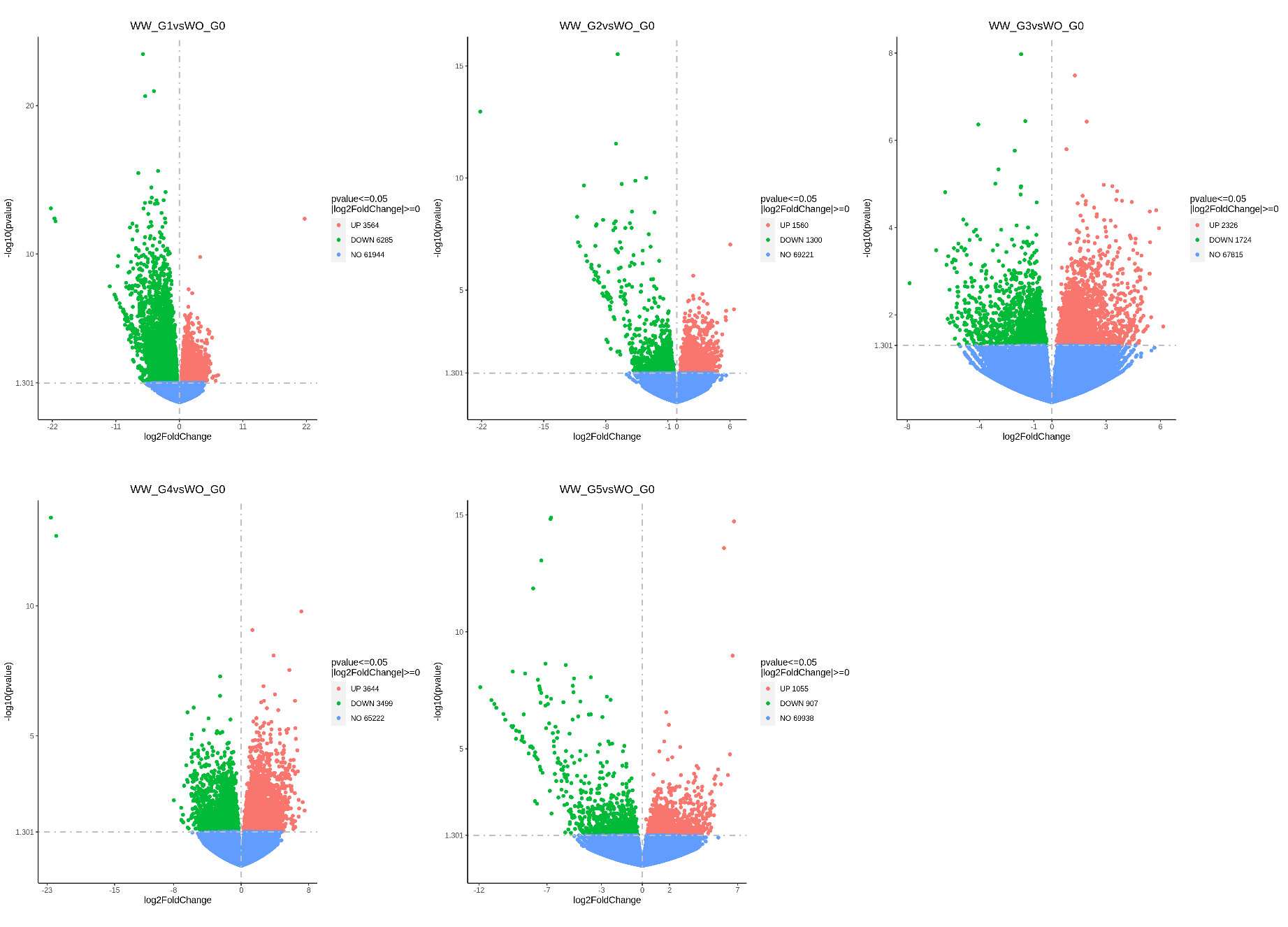
**

**eN**

**dN**

**c**

**b**

**aN**

**Supplementary Fig. 4 | Differential expression analysis of wheat-wheat treatments compared to generation 0 wheat-only**. Volcano plots illustrate upregulated (red) and downregulated (green) genes (p ≤ 0.05, |log2FoldChange| ≥ 1) relative to wheat-only control conditions. (a) generation 1, (b) generation 2, (C) generation 3, (d) generation 4, and (e) generation 5. Non-significant genes are shown in blue. The number of upregulated, downregulated, and non-significant genes is indicated in the plot legends.


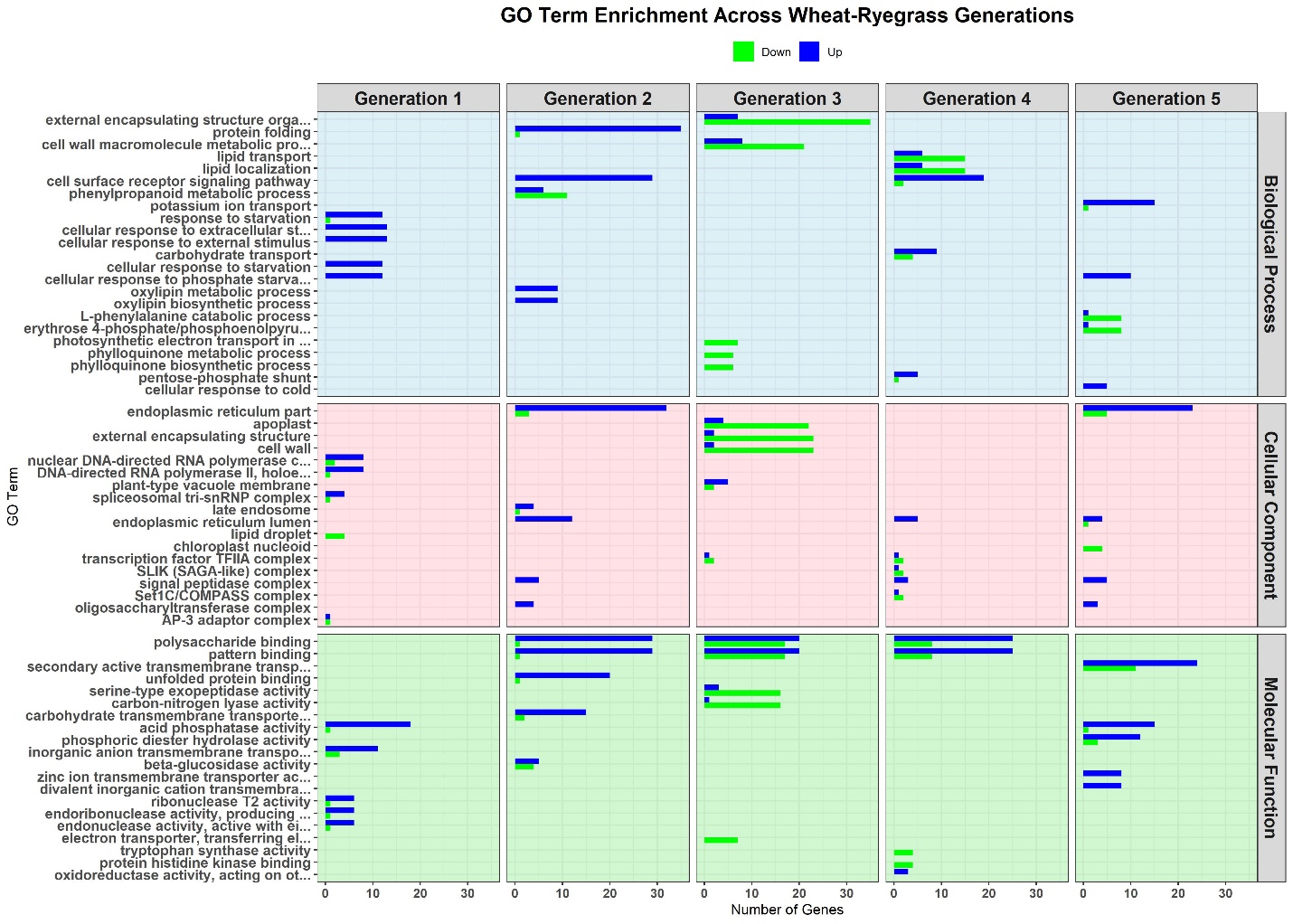


**Supplementary Fig. 5 | GO term enrichment analysis of differentially expressed genes for the wheat-ryegrass treatment**. Panels show changes in the top 10 GO terms related to biological processes (light blue), cellular components (pink), and molecular functions (light green) for each generation. Upregulated genes are represented in blue, while downregulated genes are shown in green.

**
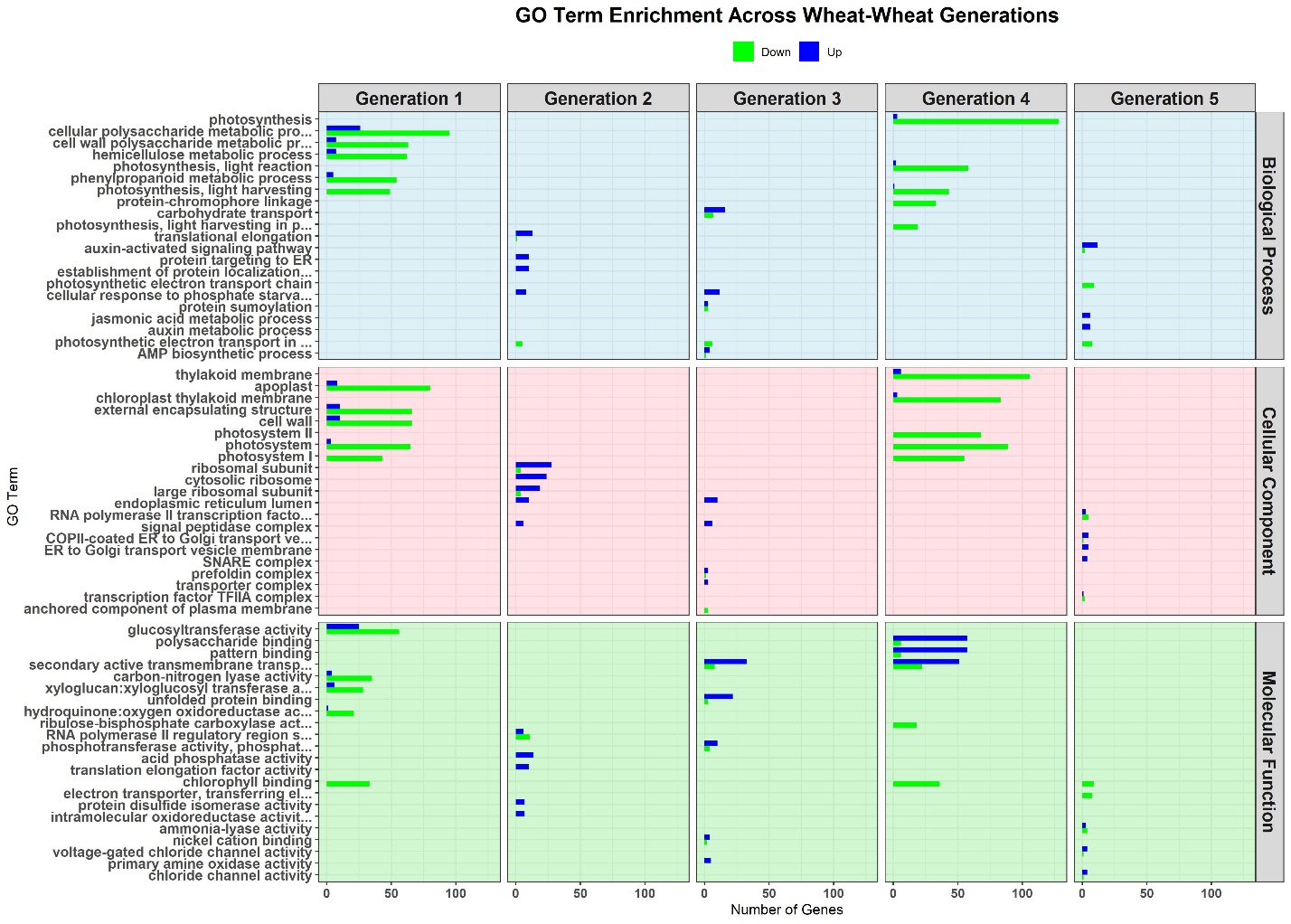
**

**Supplementary Fig. 6 | GO term enrichment analysis of differentially expressed genes for the wheat-wheat treatment**. Panels show changes in the top 10 GO terms related to biological processes (light blue), cellular components (pink), and molecular functions (light green) for each generation. Upregulated genes are represented in blue, while downregulated genes are shown in green.
